# DLK1 and DLK2, two non-canonical ligands of NOTCH receptors, differentially modulate the osteogenic differentiation of mesenchymal C3H10T1/2 cells

**DOI:** 10.1186/s40659-024-00561-7

**Published:** 2024-10-30

**Authors:** María-Milagros Rodríguez-Cano, María-Julia González-Gómez, Eva-María Monsalve, María-José M. Díaz-Guerra, Moustapha Kassem, Jorge Laborda, María-Luisa Nueda, Victoriano Baladrón

**Affiliations:** 1 Biochemistry and Molecular Biology Branch, Department of Inorganic, Organic Chemistry and Biochemistry, Pharmacy School/IB-UCLM/Biomedicine Unit, University of Castilla-La Mancha/CSIC, Albacete, Spain; 2 Biochemistry and Molecular Biology Branch, Department of Inorganic, Organic Chemistry and Biochemistry, Medical School/IB-UCLM/Biomedicine Unit, University of Castilla-La Mancha/CSIC, Albacete, Spain; 3 Biochemistry and Molecular Biology Branch, Department of Inorganic, Organic Chemistry and Biochemistry, ETSIAMB/IB-UCLM/Biomedicine Unit, University of Castilla-La Mancha/CSIC, Albacete, Spain; 4 Molecular Endocrinology Unit (KMEB), Department of Endocrinology and Metabolism, University Hospital of Odense and Danish Institute for Advanced Study, University of Southern Denmark, Odense, Denmark

**Keywords:** DLK, NOTCH, Mesenchymal C3H10T1/2 cells, Osteogenesis, ERK1/2 MAPK, p38 MAPK

## Abstract

**Background:**

C3H10T1/2 is a mesenchymal cell line capable of differentiating into osteoblasts, adipocytes and chondrocytes. The differentiation of these cells into osteoblasts is modulated by various transcription factors, such as RUNX2. Additionally, several interconnected signaling pathways, including the NOTCH pathway, play a crucial role in modulating their differentiation into mature bone cells. We have investigated the roles of DLK1 and DLK2, two non-canonical inhibitory ligands of NOTCH receptors, in the osteogenic differentiation of C3H10T1/2 cells.

**Results:**

Our results corroborate existing evidence that DLK1 acts as an inhibitor of osteogenesis. In contrast, we demonstrate for the first time that DLK2 enhances this differentiation process. Additionally, our data suggest that NOTCH2, 3 and 4 receptors may promote osteogenesis, as indicated by their increased expression during this process, whereas NOTCH1 expression, which decreases during cell differentiation, might inhibit osteogenesis. Moreover, treatment with DAPT, a NOTCH signaling inhibitor, impeded osteogenic differentiation. We have confirmed the increase in ERK1/2 MAPK and p38 MAPK phosphorylation in C3H10T1/2 cells induced to differentiate to osteoblasts. Our new findings reveal increased ERK1/2 MAPK phosphorylation in differentiated C3H10T1/2 cells with a decrease in DLK1 expression or an overexpression of DLK2, which is coincident with the behavior of those transfectants where we have detected an increase in osteogenic differentiation. Additionally, p38 MAPK phosphorylation increases in differentiated C3H10T1/2 cells with reduced DLK1 levels.

**Conclusions:**

Our results suggest that DLK1 may inhibit osteogenesis, while DLK2 may promote it, by modulating NOTCH signaling and the phosphorylation of ERK1/2 and p38 MAPK pathways. Given the established inhibitory effect of DLK proteins on NOTCH signaling, these new insights could pave the way for developing future therapeutic strategies aimed at treating bone diseases.

**Supplementary Information:**

The online version contains supplementary material available at 10.1186/s40659-024-00561-7.

## Background

Different signaling pathways collaboratively modulate the osteogenic process, including ERK1/2 MAPK, p38 MAPK, BMPs (Bone Morphogenetic Proteins), and NOTCH receptors [1], among others. In mammals, the transmembrane NOTCH protein family comprises four receptors and canonical and non-canonical ligands. Upon canonical ligand binding, NOTCH receptors undergo a first cleavage occurring at the S2 site by ADAM17/ADAM10 α-secretases [2], and the second cleavage at the S3-S4 sites by the γ-secretase complex [3]. These cleavages result in the release of the active NOTCH intracellular domain (NICD) that can translocate into the nucleus to function as a transcriptional activator by forming a complex with the CSL/RBPJκ (CBF1/supressor of hairless/Lag1/Recombination signal binding protein-Jκ) factor and co-activators such as the mastermind-like proteins 1–3 (MAML1-3). This complex activates the expression of specific target genes, including those in the *Hes/Hey* (Hairy and Enhancer-of-split homologous) family [4]. DLK (Delta-like homologous) proteins (DLK1 and DLK2) are transmembrane proteins of the NOTCH family that interact with NOTCH receptors, acting as non-canonical inhibitory ligands [5–10]. Additionally, DLK proteins are capable of self-interaction [11, 12]. Notably, the interaction between DLK1 and DLK2 results in reciprocal inhibition of each other’s activities, enhancing NOTCH1 receptor activation and signaling [9].

NOTCH signaling is reported to have a positive impact on osteogenic differentiation. Several studies suggest that activating NOTCH1 signaling can promote the differentiation of precursor cells into osteoblasts [13]. NICD1 has been shown to increase osteogenic differentiation in a dose-dependent manner [14]. Additionally, increases in the expression of *Notch1*, *Notch2*, and *Hey1* genes have been linked to the differentiation of immature osteogenic cells, and enhanced mineralization [15]. Conversely, inhibiting the NOTCH pathway has been shown to reduce the expression of osteogenic markers [16]. Furthermore, treatment with GSI (γ-secretase complex inhibitors) appears to diminish osteogenesis [17]. However, NOTCH receptor signaling is also reported to have an inhibitory impact on osteogenesis. Studies have shown that activation of NOTCH signaling or overexpression of NICD1 can impede osteogenic differentiation by the suppression of osteogenic markers like *Runx2* (Runt-related transcription factor 2), as well as hindering the mineralization of bone extracellular matrix [18]. Similarly, inhibiting NOTCH1 signaling has been observed to promote the expression of early osteogenic markers [19]. Furthermore, inhibition of NOTCH receptor signaling using DAPT, a GSI, has been shown to restore osteogenic differentiation [20].

It is proposed that the influence of NOTCH receptor signaling on osteogenesis is contingent upon the differentiation stage of the cells and the basal activation level of NOTCH signaling. It is hypothesized that early-stage activation of NOTCH signaling may hinder osteogenic differentiation by accumulation of immature osteoblasts [21]. In contrast, activation during later stages is thought to enhance osteoblast differentiation, and mineralization [21]. Consequently, whereas NOTCH2, NOTCH4, and HEY1 might support early stages of osteogenesis, NOTCH1, NOTCH3, and HES5 are believed to sustain an undifferentiated cellular state [22]. This dichotomy might also stem from differential expression of NOTCH receptor target genes. For example, HEY1, associated with late stages, is known to inhibit matrix mineralization by constraining RUNX2 activity [23], while HES1 seems to stimulate RUNX2 expression, thereby activating osteogenic differentiation [24].

Other key pathways, including ERK1/2 MAPK, have been identified as significant modulators of osteogenesis. Despite the study by Zhai and coworkers in 2017 [25] that suggests an inhibitory role in this differentiation process, the predominant evidence indicates that activation of ERK1/2 MAPK enhances osteogenic differentiation by the activation of RUNX2 [26]. Furthermore, strong evidence supports the role of p38 MAPK as an enhancer of osteogenic differentiation [27].

Current published data indicate that DLK1 may function as an inhibitor of osteogenesis. Its overexpression hinders the BMPs signaling pathway and the expression of osteogenic markers and reduces bone mineral mass. Additionally, DLK1 appears to promote osteoclastogenesis and bone resorption, by activating the NF-κB (nuclear factor Kappa-B) and increasing the synthesis of proinflammatory cytokines [28–30]. DLK1 has also been reported to suppress the expression of RUNX2 and maintain high levels of SOX9 expression, a transcription factor that biases cells towards a chondrogenic phenotype [31]. Intriguingly, treatment with anti-DLK1 antibodies has been shown to protect against bone mass loss due to estrogen deficiency [32]. The role of DLK2 in bonce cells differentiation is less understood. Recent findings [33] demonstrate that DLK2 deletion in osteoclasts inhibits osteoclast formation in vitro and contributes to a high-bone-mass phenotype in vivo. Another research indicates that DLK2 may inhibit chondrogenesis through the p38 MAPK pathway [34].

In this study, we analyze whether altered DLK1 and DLK2 expression levels affect the osteogenic differentiation process of mesenchymal C3H10T1/2 cells. Our findings reveal that while DLK1 inhibits osteogenesis, DLK2 acts as an activator of this differentiation process. This is particularly noteworthy given that DLK proteins function as inhibitors of NOTCH receptors signaling. Additionally, we examined the effects of varying DLK protein expression levels on the expression and activation of NOTCH receptors and their target genes, *Hes1* and *Hey1*. We also explored the influence of these expression levels on the phosphorylation of ERK1/2 and p38 MAPK. The insights gained from this work hold potential future applications in treating ossification disorders.

## Results

### The osteogenic differentiation of C3H10T1/2 cells in response to β-glycerophosphate, ascorbic acid, and retinoic acid (ATRA)

**Fig. 1:**
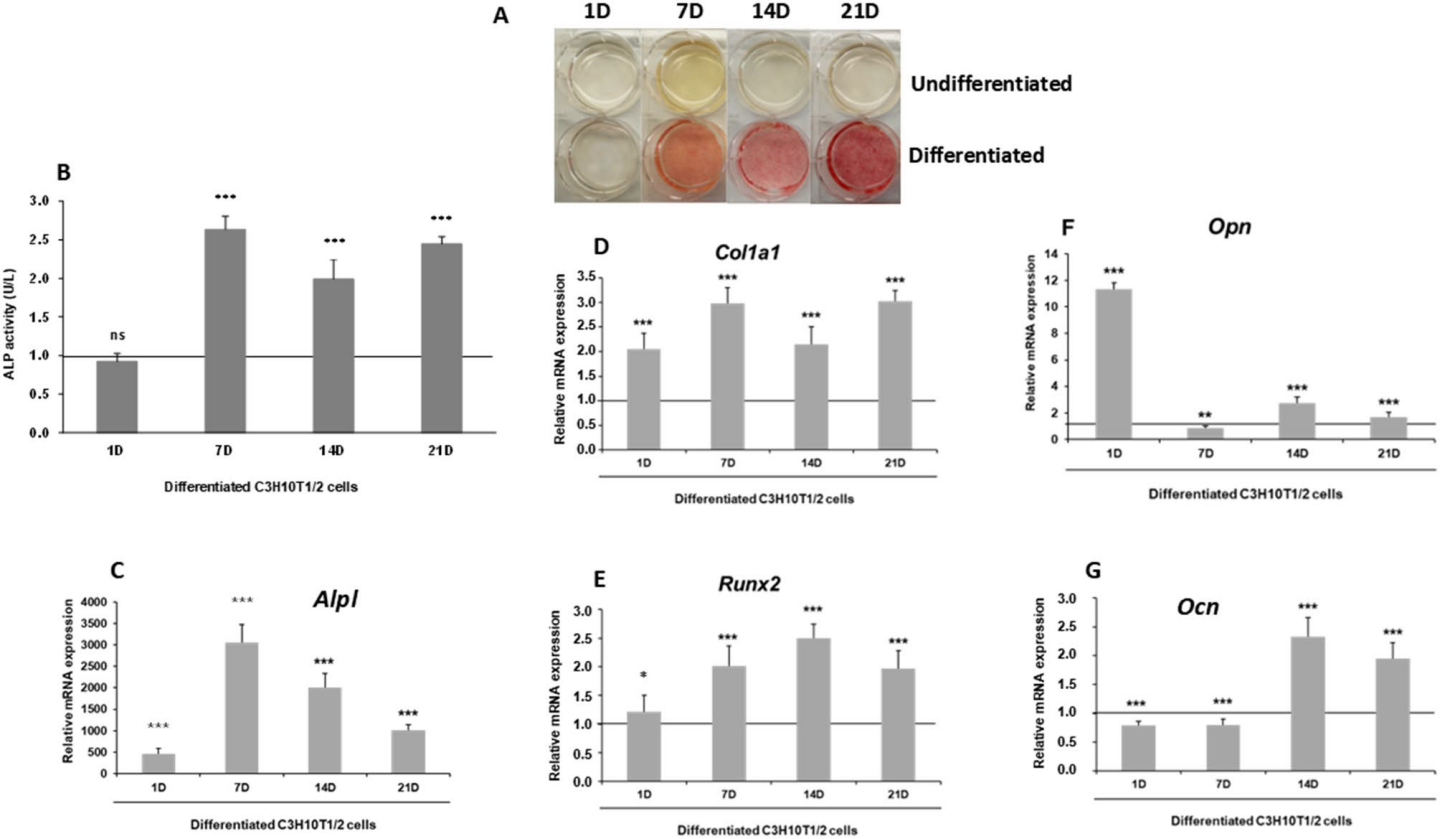
Osteogenic differentiation in C3H10T1/2 cells induced by β-glycerophosphate, ascorbic acid, and all trans retinoic acid (ATRA). **A** Alkaline phosphatase staining illustrating the progression of osteoblastic differentiation in C3H10T1/2 cells over 1, 7, 14, and 21 days of induction. **B** ALP activity (U/L) in C3H10T1/2 cells over 1, 7, 14, and 21 days of osteogenesis induction. RT-qPCR analysis of the osteogenesis-specific marker in C3H10T1/2 cells. Relative mRNA expression levels of *Alpl* (**C**) *Col1a1* (**D**), *Runx2* (**E**), *Opn* (**F**), and *Ocn* (**G**) osteogenic markers were also measured in differentiated C3H10T1/2 cells over a period of 1, 7, 14, and 21 days of induction. Expression data were normalized against the constitutive ribosomal gene *Rplp0*. Expression levels are relative to day 1 in undifferentiated cells (set arbitrarily at 1) [horizontal line]. Absence of this line in some graphs is due to overlap with the horizontal axis due to scale adjustments. Data are presented as mean ± SD from a minimum of three independent assays, each performed in triplicate. Statistical significance was assessed using Student’s *t*-test (***p ≤ 0.001, **p ≤ 0.01, and *p ≤ 0.05)

For the osteogenic differentiation of mesenchymal C3H10T1/2 cells, the literature commonly cites two primary components: β-glycerophosphate and ascorbic acid. A third component varies across studies [35]. In our experimental conditions, we utilized all-trans retinoic acid (ATRA) as this third variable component. We used the alkaline phosphatase staining method to confirm the differentiation of C3H10T1/2 cells into osteoblasts after 7, 14, and 21 days of this treatment (Fig. 1A). We observed a progressive increase in staining after 7 days of treatment. Additionally, we measured ALP activity at 1, 7, 14, and 21 days of osteogenic treatment (Fig. 1B). An increase in ALP activity was noted after 7 days, which remained consistent at 14 and 21 days. Additionally, we analyzed the expression at 1-, 7-, 14-, and 21-days post-induction of the early marker *Alpl* (alkaline phosphatase), the intermediate marker *Col1a1* (Collagen Type 1 Alpha-1 chain), *Runx2* transcription factor, and the late markers *Opn* (Osteopontin) and *Ocn* (Osteocalcin) (Fig. 1C and G). *Alpl* marker showed a rapid increase in expression, peaking on day 7, then declining by day 21 (Fig. 1C), compared with non-differentiated cells on day 1 (indicated by a horizontal line), taken as control cells. *Col1a1* expression rose from day 1 and remained elevated throughout the differentiation process expression significantly (Fig. 1D), while *Runx2* increased from day 7 onwards (Fig. 1E). Interestingly, although *Opn* is typically considered a late osteogenesis marker, it exhibited a peak in expression on the first day on the first day after induction of differentiation. This was followed by a decline over the subsequent days, dropping below the levels observed in control cells by day 7, and then rising again by day 14 (Fig. 1F). *Ocn* reached maximum expression on day 14, maintaining elevated levels at day 21 (Fig. 1G), although its expression levels on days 1 and 7 were lower than control cells.

We also focused on analyzing the expression levels of endogenous *Notch1-4* genes, their targets, *Hes1* and *Hey1*, and non-canonical genes, *Dlk1* and *Dlk2*, in both differentiated and non-differentiated C3H10T1/2 cells (Supplementary Fig. 1A–H). In undifferentiated C3H10T1/2 cells, we observed that the expression of all *Notch* genes increased with cell confluence from day 7, relative to the levels in non-differentiated cells on day 1 (horizontal line), which served as the control cells. Following osteogenic treatment, there was a decrease in *Notch1* expression compared to the control cells (Supplementary Fig. 1A), but *Notch2* (Supplementary Fig. 1B), *Notch3* (Supplementary Fig. 1C), and *Notch4* (Supplementary Fig. 1D) exhibited increased expression at all time points. In undifferentiated cells, *Hes1* expression decreased after 7 days, correlating with increased cell confluence over time in culture (Supplementary Fig. 1E). However, in cells treated with osteogenic inducers, *Hes1* expression levels increased, peaking at day 14 relative to the control cells. As for *Hey1*, its expression in undifferentiated cells rose with increasing cell confluence, reaching its maximum at day 14 compared to the control cells. Interestingly, upon osteogenic induction, *Hey1* expression also increased, achieving its highest level on day 21 of differentiation, compared to the control cells (Supplementary Fig. 1F).

Finally, in undifferentiated cells, *Dlk1* expression levels increased with cell confluence, peaking on day 14, compared to control cells (Supplementary Fig. 1G). However, when these cells were subjected to osteogenic inducers, *Dlk1* expression remained lower than the control levels. Contrasting with *Dlk1*, *Dlk2* expression was inhibited in undifferentiated cells on days 7, 14, and 21, despite increased cell confluence (Supplementary Fig. 1H). Notably, under osteogenic induction, *Dlk2* expression significantly increased on day 1, compared to the control cells.

These findings suggest that *Dlk* genes are expressed in a coordinated but inverse manner, exhibiting opposite patterns in undifferentiated cells and in cells undergoing osteogenic treatment.

### The impact of DAPT, a γ-secretase complex inhibitor,on the osteogenic differentiation of C3H10T1/2 cells

We first measured the activation of NOTCH1 and NOTCH2 receptors in differentiated cells by detecting the levels of their active intracellular domains, NICD1 and NICD2, respectively (Supplementary Fig. 2A and 2B). When global NOTCH signaling was inhibited using DAPT, we noted a reduction in the levels of both NICD1 and NICD2. These levels were normalized to the total NOTCH1 and NOTCH2 levels, respectively, and compared with non-differentiated cells on day 1 treated with DMSO (horizontal line), which was used as the control cells. The inhibitory effect in the presence of DAPT dissolved in DMSO was also observed when we analyzed the levels of global NOTCH signaling activation in C3H10T1/2 cells, as compared with cells in the presence of DMSO, as evidenced by luciferase assays (Supplementary Fig. 2C). A positive control of luciferase activity by transfecting cells with plasmid pNICD1, which express an active form of the NOTCH1 receptor, compared with cells transfected with an empty vector (V), is also shown (Supplementary Fig. 2D). We also evaluated the expression of two NOTCH receptor target genes, *Hes1* and *Hey1*, in C3H10T1/2 cells undergoing osteoblast differentiation, both with and without DAPT treatment (Supplementary Fig. 2E and 2 F, respectively). Data normalization was performed against non-differentiated cells on day 1 treated with DMSO (horizontal line), and the expression levels of each gene were compared to those in similarly timed differentiated cells treated with DMSO. We found that the expression level of *Hes1* increased on days 7 and 14 with DMSO compared to control cells (Supplementary Fig. 2C). However, *Hes1* expression decreased in the presence of DAPT (days 1, 7, and 14), relative to control cells. Conversely, while *Hey1* expression also increased on days 7 and 14 with DMSO (Supplementary Fig. 2D), DAPT treatment did not significantly alter *Hey1* expression levels in differentiated cells, except for a noted inhibition on day 21 of treatment.

**Fig. 2:**
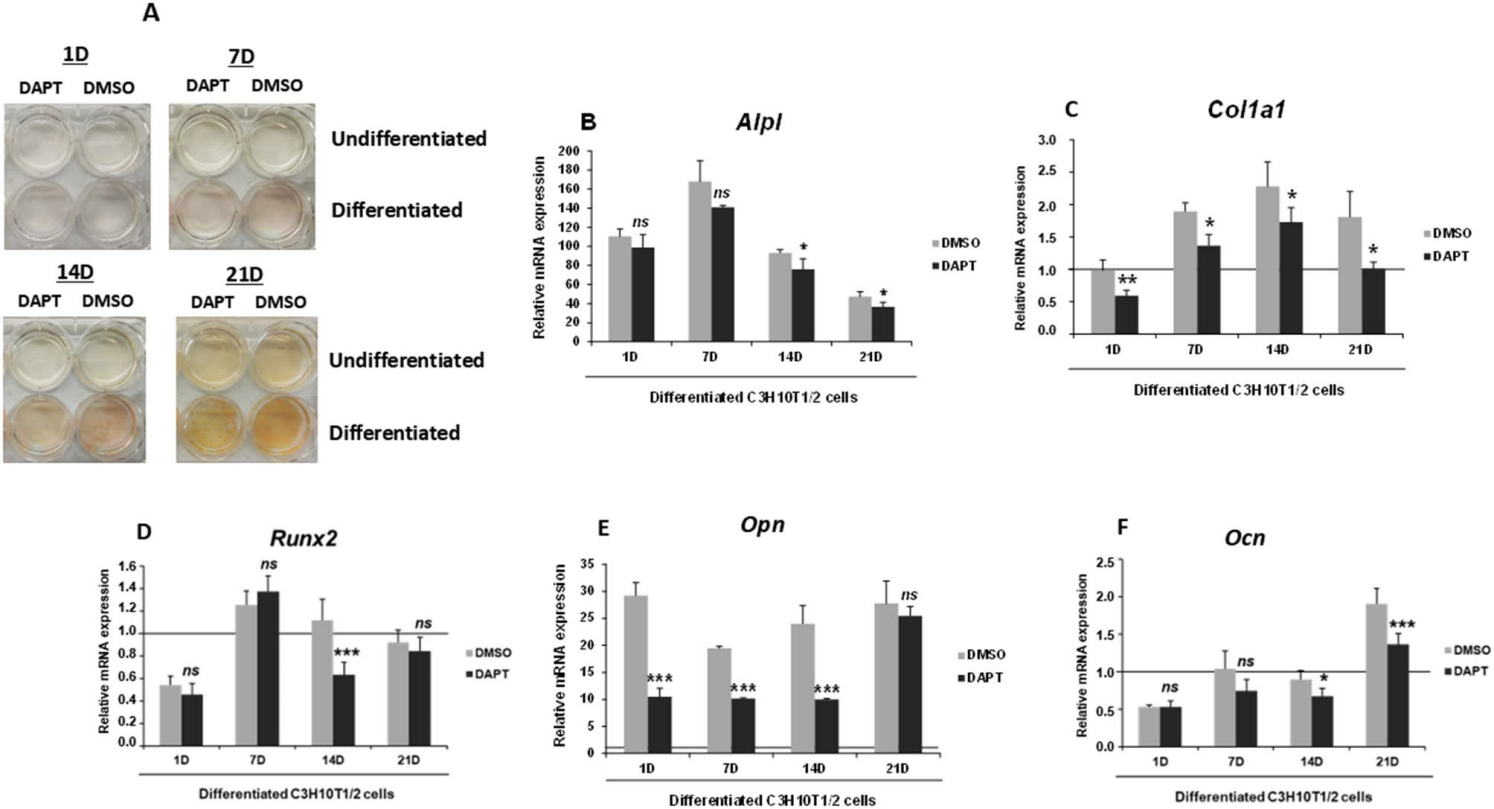
Influence of DAPT, a γ-secretase complex inhibitor, on the expression of osteogenic markers during the differentiation of C3H10T1/2 cells . **A** Representative images from alkaline phosphatase staining depict the contrast in osteogenic activity of undifferentiated and differentiated C3H10T1/2 cells over 1, 7, 14, and 21 days [D], in both the presence and absence of the DAPT inhibitor. **B**–**F** The figure further includes a RT-qPCR analysis of the relative mRNA expression levels of osteogenic markers *Alpl* (**B**), *Col1a1* (**C**), *Runx2* (**D**), *Opn* (**E**), and *Ocn* (**F**) in C3H10T1/2 cells undergoing osteoblastic differentiation with or without DAPT treatment at the same time points. The RT-qPCR data were normalized against the mRNA levels of the constitutive ribosomal gene *Rplp0*, and expression levels were calculated relative to day 1 in undifferentiated cells treated with DMSO (horizontal line). The absence of the horizontal line in some graphs is due to its overlap with the horizontal axis because of the vertical axis scale. Data are presented as mean ± SD from at least three independent experiments, each performed in triplicate. Statistical significance was determined using Student’s *t*-test (***p ≤ 0.001, **p ≤ 0.01, and *p ≤ 0.05), with non-significant results indicated as ns

Then, we analyzed the impact of DAPT’s inhibitory effect on global NOTCH signaling, on the osteogenic differentiation of C3H10T1/2 cells by employing the alkaline phosphatase staining method. As depicted in Fig. 2A, adding DAPT to non-differentiated cells did not result in any significant change in alkaline phosphatase staining at any of the analyzed time points, compared to the control cells treated with DMSO. However, when DAPT was included in the osteogenic differentiation cocktail, a noticeable decrease in alkaline phosphatase staining was observed at all time points.

Finally, we evaluated the changes in the expression levels of osteogenic differentiation markers in C3H10T1/2 cells treated with DAPT (Fig. 2B–F). Data were again normalized to non-differentiated cells on day 1 of culture (horizontal line), and the expression of each marker was compared with values from DMSO-treated differentiated cells, serving as control cells. As shown in Fig. 2B, DAPT did not affect *Alpl* expression on days 1 and 7. However, its expression was significantly inhibited by DAPT on days 14 and 21, compared to control cells. The expression levels of *Col1a1* were consistently lower in the DAPT-treated cells across all analyzed time points of osteogenic differentiation, indicating significant inhibition (Fig. 2C). The expression pattern of *Runx2* (Fig. 2D) mostly mirrored that of the control group, except for a marked reduction on day 14. The expression of *Opn* was reduced during days 1, 7, and 14 of osteogenic treatment when compared with control cells (Fig. 2E). Lastly, *Ocn* expression levels were similar to control cells on days 1 and 7 but showed notable inhibition on days 14 and 21 (Fig. 2F).

### Generation of stable transfectant pools for *Dlk1* and *Dlk2* in C3H10T1/2 cells

**Fig. 3:**
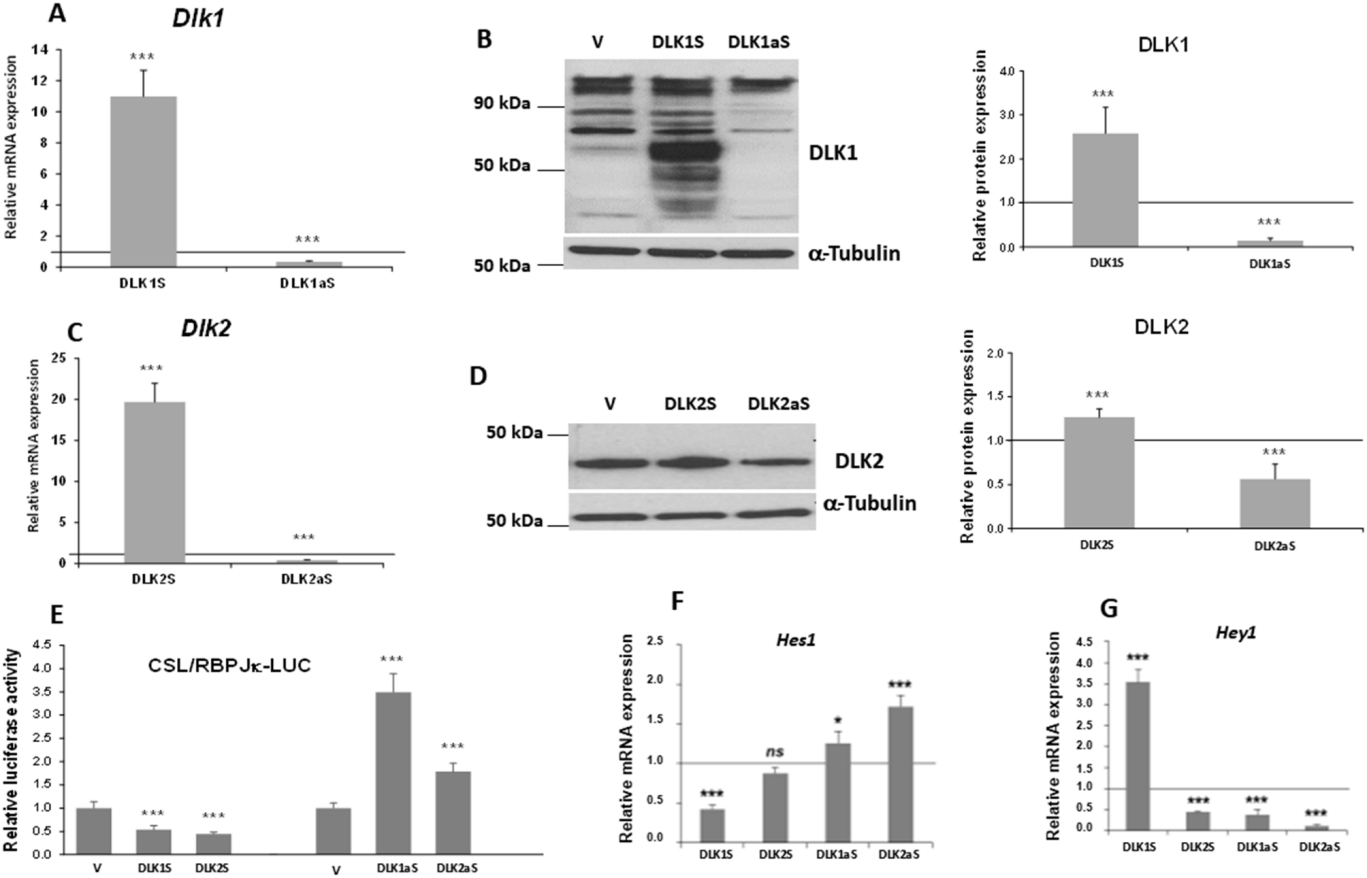
Characterization of *Dlk1* and *Dlk2* stable transfectant pools in C3H10T1/2 cells. RT-qPCR analysis of the relative mRNA expression levels of *Dlk1* in DLK1S and DLK1aS stable transfectant pools (**A**), and *Dlk2* in DLK2S and DLK2aS stable transfectant pools (**B**). **C**, **D** Representative Western blots and densitometric analyses illustrate DLK1 (50–60 kDa) (**C**) and DLK2 (40 kDa) (**D**) protein expression levels in these pools. **E** Global NOTCH signaling activity assessed by luciferase assay in DLK1 and DLK2 transfectants compared to control cells transfected with empty vector (V). The figure also presents RT-qPCR analysis of *Hes1* (**F**) and *Hey1* (**G**) mRNA expression levels in each stable transfectant pool. α-Tubulin was employed as a control for loading and sample quality in Western blot assays. Data from RT-qPCR assays were normalized against the constitutive ribosomal gene *Rplp0*, with expression levels calculated relative to cells stably transfected with the corresponding empty vector (set arbitrarily at 1) [horizontal line]. Data are presented as mean ± SD from a minimum of three independent assays performed in triplicate. Statistical significance was assessed using Student’s *t*-test (***p ≤ 0.001,  and *p ≤ 0.05), and non-significant results are denoted as ns

Given their roles as NOTCH signaling inhibitors and their contrasting expression profiles during osteogenic differentiation in C3H10T1/2 cells, we aimed to generate stable transfectant pools of *Dlk1* and *Dlk2* genes in C3H10T1/2 cells that either overexpress or exhibit reduced expression levels of these genes (Fig. 3). We confirmed that the stable transfections resulted in the expected changes at both mRNA (Fig. 3A and C) and protein levels (Fig. 3B and D), in comparison to cells stably transfected with empty vectors, which served as control cells. These changes at the protein level aligned with the levels of activation of global NOTCH signaling in DLK transfected cells, as evidenced by luciferase assays (Fig. 3E).

We also observed notable effects of DLK protein expression levels on the endogenous expression of *Hes1* and *Hey1* (Fig. 3F and G), compared to control cells. Specifically, stable overexpression of *Dlk1* led to a decrease in *Hes1* expression, while *Dlk2* overexpression did not significantly alter *Hes1* levels (Fig. 3F). Conversely, when the expression of both *Dlk1* and *Dlk2* was downregulated, there was an activation of *Hes1* expression (Fig. 3F). Interestingly, overexpressing DLK1 unexpectedly increased *Hey1* expression, whereas cells overexpressing DLK2 showed a significant reduction in *Hey1* levels (Fig. 3G). Additionally, a decrease in the expression of both *Dlk* genes surprisingly resulted in reduced *Hey1* expression (Fig. 3G).

We also observed that the expression levels of DLK proteins in undifferentiated C3H10T1/2 cells influenced the endogenous expression of *Notch* (Supplementary Fig. 3A–D), in comparison to control cells. Specifically, the overexpression of *Dlk1* or reduced expression of *Dlk2* led to an increase in the expression of all four *Notch* genes, with a particularly notable effect on *Notch1*. Conversely, the overexpression of *Dlk2* or reduced expression of *Dlk1* elevated the expression levels of *Notch2*, *Notch3*, and *Notch4* but no significative changes were observed in *Notch1* expression.

Additionally, we observed that overexpression of *Dlk1* in undifferentiated cells resulted in a decrease in *Dlk2* expression, while reduced expression of *Dlk1* led to an increase in *Dlk2* expression (Supplementary Fig. 3E). In contrast, while overexpressing *Dlk2* raised *Dlk1* expression levels, decreasing *Dlk2* expression did not significantly impact *Dlk1* expression (Supplementary Fig. 3F).

### Analysis of the expression levels of osteogenic markers, as well as *Notch*, *Hes1,* and *Hey1 *genes, in *Dlk1* and *Dlk2* stable transfectant pools of C3H10T1/2 cells induced to differentiate into osteoblasts

**Fig. 4:**
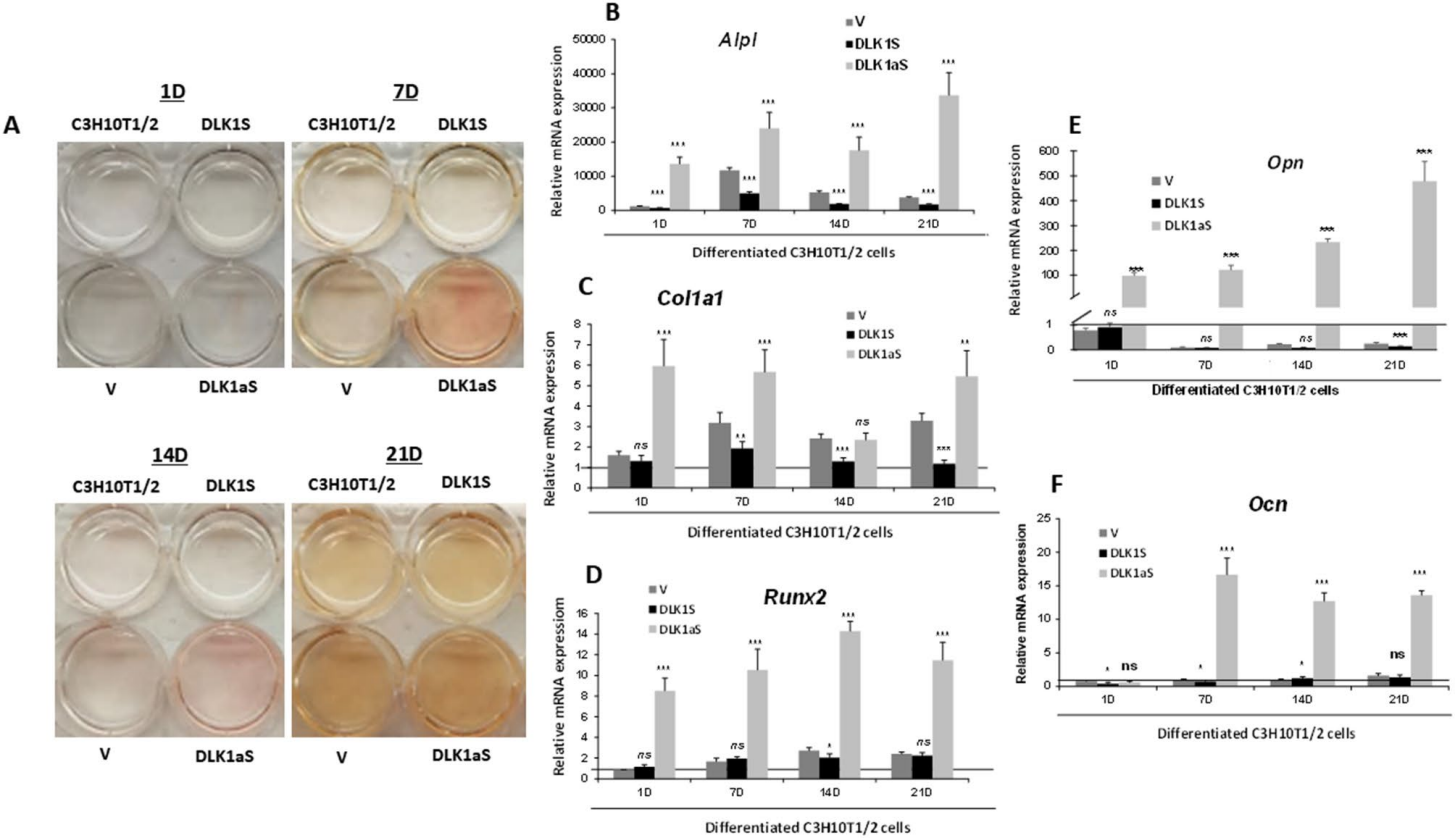
Alkaline phosphatase staining and analysis of osteogenic marker expression levels in *Dlk1* stable transfectant pools of C3H10T1/2 cells. **A** This part showcases representative images of alkaline phosphatase staining in cell culture wells. It includes both non-transfected and *Dlk1* stable transfectant pools of C3H10T1/2 cells that have undergone osteoblastic differentiation. The images capture the staining results at 1-, 7-, 14-, and 21-days [D] post-induction of osteogenic differentiation. The cultures include C3H10T1/2 non-transfected cells, cells transfected with empty vector control [V], and cells from the DLK1S and DLK1aS transfectant pools, providing a comparative view of alkaline phosphatase activity across different genetic modifications and stages of differentiation. In this figure, we also show a RT-qPCR analysis of the relative mRNA expression levels of key osteogenic markers in *Dlk1* sense (DLK1S) and antisense (DLK1aS) stable transfectant pools of C3H10T1/2 cells. The markers analyzed include *Alpl* (**B**), *Col1a1* (**C**), *Runx2* (**D**), *Opn* (**E**), and *Ocn* (**F**). The expression levels were measured in cells differentiated into osteoblasts over a period of 1-, 7-, 14-, and 21-days [D] post-induction of osteogenic differentiation. The RT-qPCR data were normalized against the mRNA levels of the constitutive ribosomal gene *Rplp0*, with expression levels calculated relative to day 1 in cells stably transfected with the empty vector (set arbitrarily at 1) [horizontal line]. The absence of the horizontal line in some graphs is due to its overlap with the horizontal axis resulting from the vertical axis scale adjustments. Results are shown as mean ± SD from a minimum of three independent assays, each performed in triplicate. Statistical significance was assessed using Student’s *t*-test (***p ≤ 0.001, **p ≤ 0.01, and *p ≤ 0.05), and non-significant results are denoted as ns

**Fig. 5:**
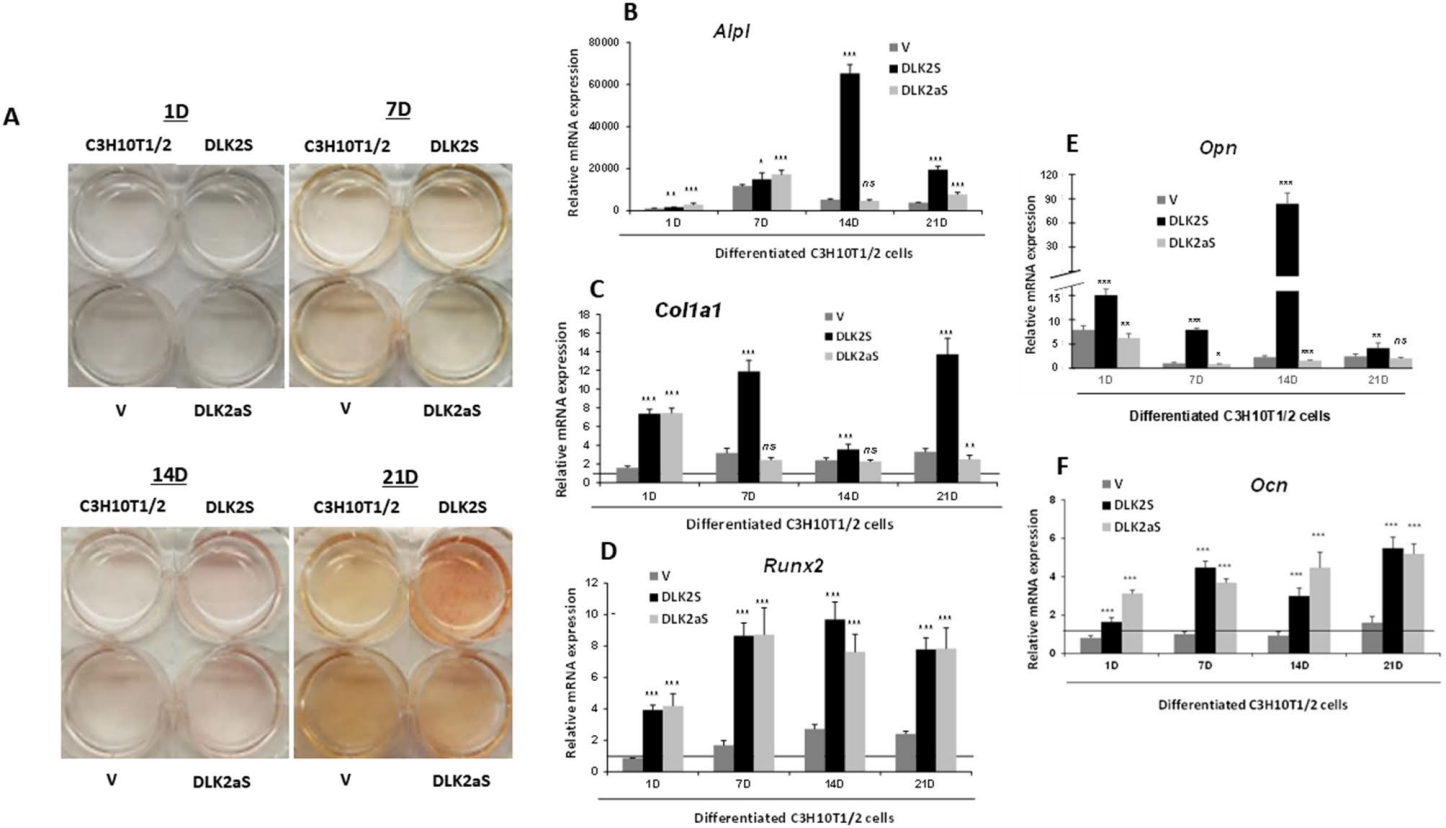
Alkaline phosphatase staining and analysis of osteogenic marker expression levels in *Dlk2* stable transfectant pools of C3H10T1/2 cells. **A** This part showcases representative images of alkaline phosphatase staining in cell culture wells. It includes both transfected and *Dlk2* stable transfectant pools of C3H10T1/2 cells that have undergone osteoblastic differentiation. The images capture the staining results at 1-, 7-, 14-, and 21-days [D] post-induction of osteogenic differentiation. The cultures include C3H10T1/2 non-transfected cells, cells transfected with empty vector control [V], and cells from the DLK2S and DLK2aS transfectant pools, providing a comparative view of alkaline phosphatase activity across different genetic modifications and stages of differentiation. In this figure, we also show a RT-qPCR analysis of the relative mRNA expression levels of key osteogenic markers in *Dlk2* sense (DLK2S) and antisense (DLK2aS) stable transfectant pools of C3H10T1/2 cells. The markers analyzed include *Alpl* (**B**), *Col1a1* (**C**), *Runx2* (**D**), *Opn* (**E**), and *Ocn* (**F**). The expression levels were measured in cells differentiated into osteoblasts over a period of 1-, 7-, 14-, and 21-days [D] post-induction of osteogenic differentiation. The RT-qPCR data were normalized against the mRNA expression levels of the ribosomal gene *Rplp0*. Expression levels for each marker were compared with values from cells stably transfected with the empty vector on day 1, set as a baseline (horizontal line). The absence of the horizontal line in some graphs is due to its overlap with the horizontal axis, resulting from the scaling of the vertical axis. Data are presented as mean ± SD from at least three independent assays, each performed in triplicate. Statistical significance was evaluated using Student’s *t*-test (***p ≤ 0.001, **p ≤ 0.01, and *p ≤ 0.05), and non-significant results are indicated as ns

We conducted osteogenesis assays on *Dlk* stable transfectant pools of C3H10T1/2 cells, assessing first the level of osteogenic differentiation using the alkaline phosphatase staining method (Figs. 4A and 5A). Our observations revealed enhanced osteogenic differentiation in cells where DLK2 was overexpressed or DLK1 expression was reduced, in comparison to cells transfected with an empty vector or non-transfected C3H10T1/2 cells. These results indicate that DLK1 acts as an inhibitor, while DLK2 functions as an activator in the osteogenic differentiation process of C3H10T1/2 cells.

We further assessed the expression levels of osteogenic markers in *Dlk* stable transfectant pools by using RT-qPCR (Figs. 4B and F and 5B and F), at 1, 7, 14, and 21 days following the induction of osteogenic differentiation. Data normalization was performed against values from non-differentiated cells on day 1 of culture (horizontal line), and expression levels of each marker were compared with those from differentiated cells stably transfected with the corresponding empty vector, serving as control cells.

Among the transfectant pools, DLK1aS and DLK2S showed notable increases in the expression of *Alpl*, compared to control cells, with peak expressions on days 7 (Fig. 4B) and 14 (Fig. 5B) of induction, respectively. The DLK2aS transfectant pool (Fig. 5B) displayed a slightly higher expression of *Alpl* than control cells, reaching its maximum on day 7. In contrast, the DLK1S transfectant pool (Fig. 4B) consistently exhibited lower *Alpl* expression levels compared to control cells throughout the differentiation process. The DLK1S pool (Fig. 4C) consistently exhibited *Col1a1* expression levels that were equal to or lower than those of control cells throughout the differentiation process. A similar trend was seen in the DLK2aS pool (Fig. 5C), except for a significant increase on day 1 of differentiation. On the other hand, both the DLK1aS (Fig. 4C) and DLK2S (Fig. 5C) transfectant pools showed higher *Col1a1* expression levels compared to control cells, except on day 14, where DLK1aS exhibited similar expression levels to the controls. Notably, the DLK1aS (Fig. 4D), DLK2S (Fig. 5D), and DLK2aS (Fig. 5D) transfectant pools exhibited a significant increase in *Runx2* expression. Among these, the DLK1aS pool showed the highest expression levels, peaking on day 14 post-induction. The DLK2aS pool reached its maximum *Runx2* expression earlier, on day 7 of differentiation, whereas DLK2S attained its peak on day 14. In contrast, the *Runx2* expression level in the DLK1S transfectant pool (Fig. 4D) was consistently similar to or even lower than that observed in control cells. Regarding the *Opn* marker, DLK1S and DLK2aS pools consistently demonstrated lower expression levels than control cells, while DLK2S and DLK1aS pools experienced a significant increase, especially noticeable in the DLK1aS pool (Figs. 4E and 5E). The highest expression of *Opn* in the DLK2S pool occurred on day 14 of differentiation, and in the DLK1aS pool, it happened on day 21. We found that the DLK2S, DLK1aS, and DLK2aS stable transfectant pools exhibited high expression levels of *Ocn*, with DLK1aS cells showing particularly elevated values (Figs. 4F and 5F). The DLK2S and DLK2aS pools (Fig. 5F) reached their peak *Ocn* expression on day 21 of differentiation, whereas the DLK1aS pool (Fig. 4F) displayed increased levels starting from day 7. In contrast, the DLK1S pool (Fig. 4F) showed *Ocn* expression levels similar to those of control cells. These results lead us to conclude that while both DLK1 and DLK2 proteins are inhibitors of NOTCH receptor signaling, DLK1 inhibits and DLK2 potentiates osteogenesis.

Given the contrasting effects of DLK1 and DLK2 proteins on the osteogenesis process in C3H10T1/2 cells, despite both proteins are NOTCH signaling inhibitors, we were motivated to investigate how varying levels of DLK1 and DLK2 in these transfectant pools influence the expression of *Notch* genes and their targets, *Hes1* and *Hey1*, during osteoblast differentiation (Supplementary Figs. 4 and 5). We normalized data against the values from non-differentiated cells on day 1 of culture (horizontal line). For each marker, expression levels were compared to those of differentiated cells stably transfected with the corresponding empty vector, which served as control cells.

Notably, all transfectant pools experienced a marked decrease in *Notch1* expression on the first day of osteogenic differentiation, particularly evident in the DLK1aS (Supplementary Fig. 4A) and DLK2S (Supplementary Fig. 5A) pools. Regarding *Notch2*, the DLK1S and DLK1aS pools (Supplementary Fig. 4B) showed the highest expression levels compared to controls. The DLK2S and DLK2aS pools (Supplementary Fig. 5B) also had elevated *Notch2* expression, with the DLK2aS pool being notably higher. For *Notch3*, all transfectant pools exhibited expression levels equal to or surpassing those of control cells (Supplementary Fig. 4 C and 5 C). The DLK1aS pool had the highest expression, closely followed by DLK2S. The DLK1S and DLK2aS pools showed *Notch3* levels similar to controls. Finally, in the case of *Notch4*, the DLK1aS (Supplementary Fig. 4D) and DLK2S (Supplementary Fig. 5D) pools demonstrated higher expression than control cells, with DLK2S having the most pronounced increase. The DLK1S and DLK2aS pools displayed *Notch4* levels equal to or lower than control cells.

As indicated in Supplementary Fig. 4E and 5E, the *Hes1* expression level increased during osteogenic differentiation across all transfectant pools compared to control cells, with the DLK2S pool (Supplementary Fig. 5E) showing the highest *Hes1* expression. Conversely, *Hey1* expression was highest in the DLK1S cells, increasing during osteogenic differentiation, while it was lower in DLK1aS cells compared to controls (Supplementary Fig. 4F). In the DLK2S and DLK2aS pools, *Hey1* expression was lower than in control cells (Supplementary Fig. 5F).

These findings suggest that DLK1 and DLK2 have differential effects on the expression of *Notch* genes and their targets during osteogenic differentiation. This difference may partially account for the opposing impacts of DLK1 and DLK2 on osteogenesis as observed in our study.

### Analysis of kinase signaling pathways involved in the osteogenesis of C3H10T1/2 cells 

We explored potential interactions between DLK proteins and some key kinase signaling pathways involved in osteogenesis. We first conducted osteogenesis assays in non-transfected C3H10T1/2 cells and used the alkaline phosphatase staining method to assess the effects of inhibiting ERK1/2 MAPK, PI3K/AKT, mTOR (mammalian Target of Rapamycin), and p38 MAPK signaling pathways on osteogenic differentiation at 1, 7, 14, and 21 days (Supplementary Fig. 6). Our findings revealed that the addition of U0126, an ERK1/2 MAPK inhibitor, led to a significant reduction in osteogenic activity compared to control cells, as indicated by alkaline phosphatase staining. Interestingly, treatment with the mTOR inhibitor rapamycin resulted in greater staining than control cells. The PI3K/AKT pathway inhibitor LY294002 yielded staining similar to controls. Lastly, inhibiting p38 MAPK signaling with SB203580 significantly reduced staining compared to controls. Our findings demonstrated that ERK1/2 MAPK and p38 MAPK pathways enhance osteogenic differentiation in C3H10T1/2 cells, while mTOR kinase serves as an inhibitor and PI3K/AKT may be less involved.

We observed that phosphorylation of ERK1/2 increased throughout the osteogenic process of non-transfected C3H10T1/2 cells compared to the baseline on day 1 in undifferentiated cells, which we used as control cells (Supplementary Fig. 7A). The peak phosphorylation level was reached on day 14 post-induction of osteogenic treatment. We next analyzed the phosphorylation levels of this kinase in DLK1 and DLK2 stable transfectants during osteogenic differentiation. Data were normalized against non-differentiated cells on day 1 of culture (horizontal line), and phosphorylation levels were compared with those of differentiated cells stably transfected with the corresponding empty vector, serving as control cells.

**Fig. 6:**
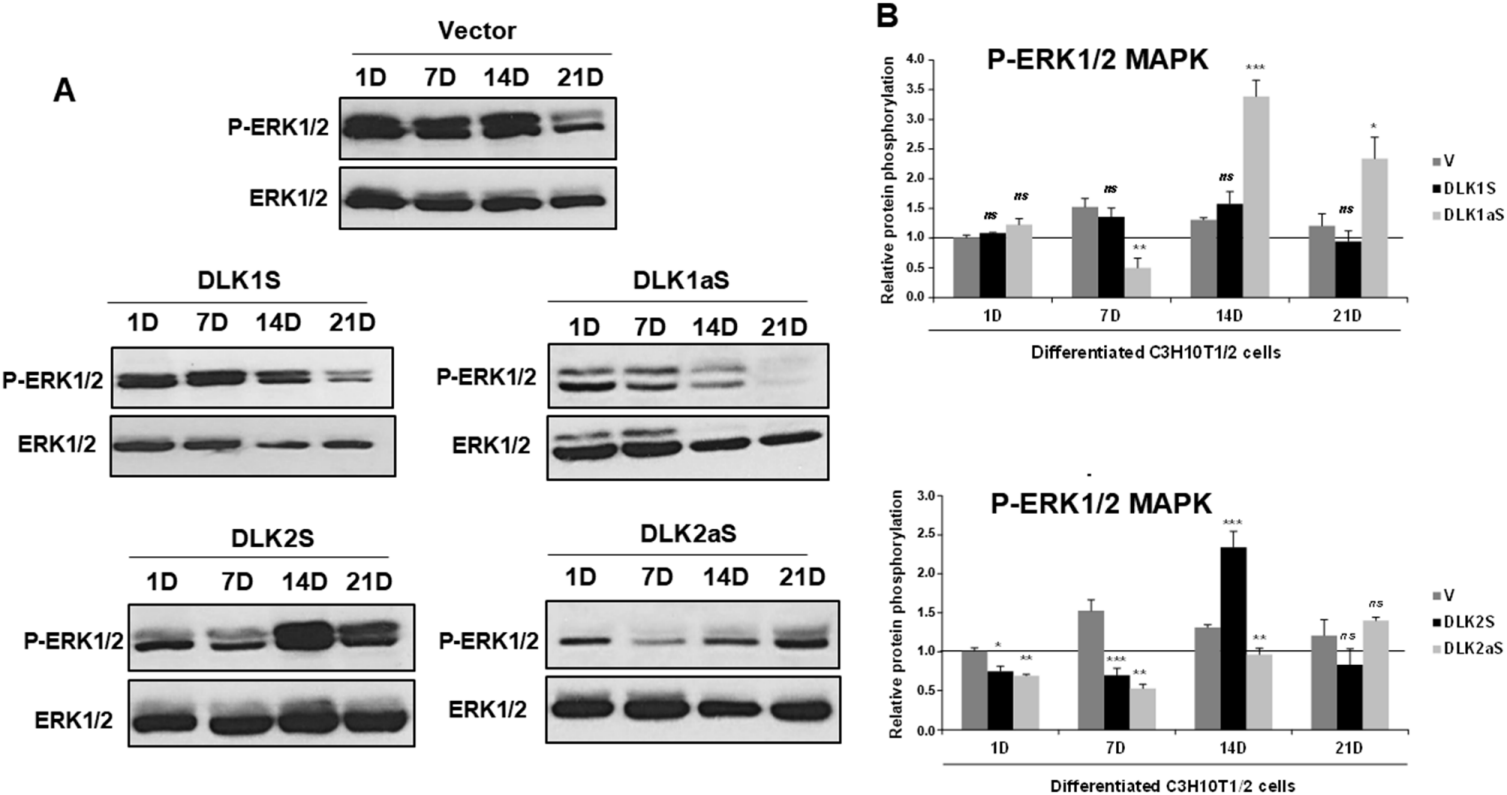
ERK1/2 MAPK phosphorylation dynamics in *Dlk1* and *Dlk2* stable transfectant pools during osteogenic differentiation. **A** Representative Western blot images displaying the phosphorylation levels of ERK1/2 MAPK (42–44 kDa) in *Dlk1* and *Dlk2* stable transfectant pools of C3H10T1/2 cells at various stages of osteogenic differentiation (1, 7, 14, and 21 days [D]). **B** Densitometric analysis quantifying ERK1/2 MAPK phosphorylation levels in *Dlk1* and *Dlk2* transfectants during osteogenic differentiation. The data are normalized to total ERK1/2 MAPK expression, serving as control for loading and sample integrity. The baseline phosphorylation level was set using data from non-differentiated cells stably transfected with the empty vector on day 1 (indicated by the horizontal line). The densitometric results are presented as the mean ± SD from at least three independent experiments, each performed in triplicate. Statistical analysis was conducted using Student’s *t*-test to compare each transfectant pool at different time points against the day 1 baseline, with significance levels marked as ***p ≤ 0.001, **p ≤ 0.01, and *p ≤ 0.05. Results not reaching statistical significance are denoted as ns

We then analyzed the phosphorylation levels of ERK1/2 MAPK in all DLK protein stable transfectants throughout the osteogenic differentiation process (Fig. 6A and B). In the early phase (days 1 and 7), in DLK2 transfectants, and on day 7, in the DLK1aS transfectant, ERK1/2 MAPK phosphorylation levels decreased compared to controls. DLK1S transfectants showed similar levels to controls during these days. However, by day 14, an increase in ERK1/2 MAPK phosphorylation was noted in both DLK1aS and DLK2S transfectants, while DLK1S transfectant did not exhibit significant variations. The DLK2aS transfectant displayed lower phosphorylation levels than control cells. By day 21, most transfectants showed no significant changes in phosphorylation levels compared to controls, except for the DLK1aS transfectant, which maintained elevated phosphorylation levels. These findings suggest that the ERK1/2 MAPK kinase is actively involved in the osteogenic process of these cells and its activity may be modulated by the levels of DLK proteins.

**Fig. 7:**
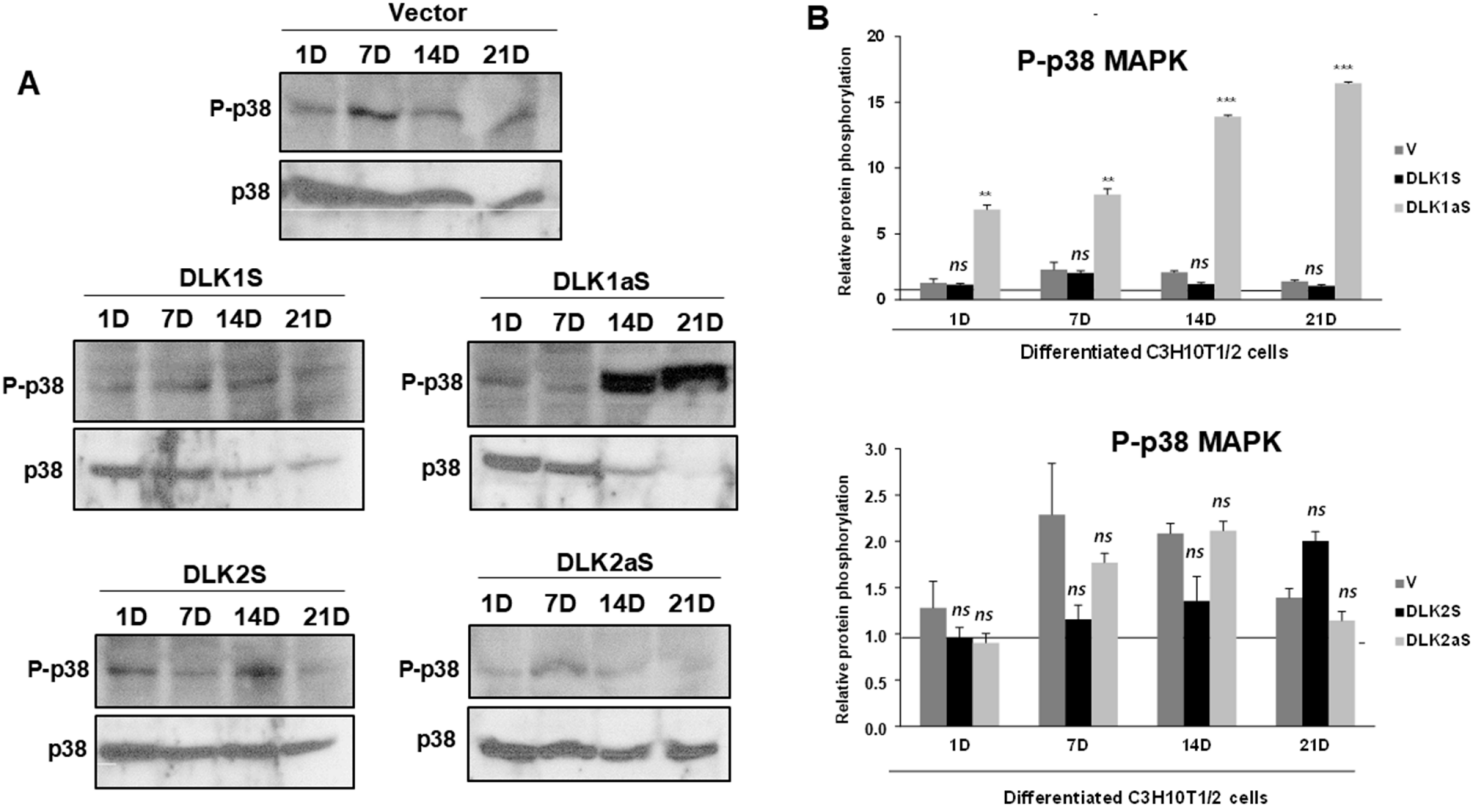
p38 MAPK phosphorylation dynamics in *Dlk1* and *Dlk2* stable transfectant pools during osteogenic differentiation. **A** Representative Western blot images showcase the phosphorylation levels of p38 MAPK phosphorylation in *Dlk1* and *Dlk2* stable transfectant pools of C3H10T1/2 cells at various stages of osteogenic differentiation (1, 7, 14, and 21 days [D]). **B** Densitometric analysis provides a quantitative assessment of p38 MAPK phosphorylation in DLK1 and DLK2 transfectant pools. The data are normalized to total p38 MAPK expression, serving as controls for loading and sample integrity. The baseline phosphorylation level was set using data from non-differentiated cells stably transfected with the empty vector on day 1 (indicated by the horizontal line). The densitometric results are presented as the mean ± SD from at least three independent experiments, each performed in triplicate. Statistical analysis was conducted using Student’s *t*-test to compare each transfectant pool at different time points against the day 1 baseline, with significance levels marked as ***p ≤ 0.001, and **p ≤ 0.01. Results not reaching statistical significance are denoted as ns

Our findings also indicated a progressive increase in the phosphorylation of p38 MAPK, peaking on day 7 of differentiation during the osteogenic differentiation of non-transfected C3H10T1/2 cells (Supplementary Fig. 7B). We then analyzed the phosphorylation levels of p38 MAPK in all DLK protein stable transfectants throughout the osteogenic differentiation process (Fig. 7A and B). For consistency, data were normalized against non-differentiated cells on day 1 of culture (horizontal line), and phosphorylation levels were compared to those in differentiated cells transfected with the corresponding empty vector, used as control cells. Our results revealed that in transfectants with decreased *Dlk1* expression, there was an increase in p38 MAPK phosphorylation across the entire differentiation period. In contrast, the other DLK stable transfectants, including those overexpressing *Dlk1* and *Dlk2*, did not exhibit significant differences in p38 MAPK phosphorylation levels during osteogenic differentiation.

## Discussion

We successfully induced osteoblastic differentiation in C3H10T1/2 cells over a 21-day treatment period. This induction was confirmed through alkaline phosphatase (ALP) staining, ALP activity assays, and the analysis of specific osteogenic marker expression levels. ALP staining in cell cultures showed a progressive increase after 7 days of treatment. ALP activity increased markedly at 7 days and remained consistent at 14 and 21 days of the osteogenic differentiation process. Similarly, the analysis of *Alpl* mRNA expression levels revealed an increase on day 7, which gradually decreased thereafter.

Intriguingly, *Opn* also displayed a significant expression peak on day 1 of differentiation. This unexpected surge might be linked to *Opn*’s role in inhibiting cell proliferation at the early stages of osteogenic differentiation, thereby facilitating the differentiation process [36].

As expected, we observed that *Dlk1* expression increased with cell confluence in culture of non-induced C3H10T1/2 cells, whereas *Dlk2* expression decreased. Intriguingly, this trend reversed during osteogenesis. *Dlk1* expression significantly decreased compared to undifferentiated cells, while *Dlk2* expression markedly increased during osteogenesis. These findings suggest a coordinated yet opposite behavior of these genes in both differentiated and non-differentiated cells, corroborating our previous research [9, 37] that implies that successful osteogenic differentiation may require a decrease in *Dlk1* expression and an increase in *Dlk2* expression. These results lend further support to existing evidence that positions DLK1 as an inhibitor of osteogenic differentiation in mesenchymal cells [28, 32, 38]. These results may suggest, for the first time, a potential role for DLK2 as an enhancer of this differentiation process in C3H10T1/2 cells.

We aimed to discern whether these proteins affect osteogenesis of the C3H10T1/2 mesenchymal cell line through NOTCH receptor signaling and the activity of kinases known to be involved in osteogenesis that are interconnected with NOTCH signaling. The role of NOTCH signaling in osteogenesis is more extensively documented, with studies suggesting that NOTCH signaling facilitates this process [13, 15, 16], and others describing that this signaling pathway exerts an inhibitory role [18–20]. The opposite effects of NOTCH signaling on osteogenesis may be due to the differentiation stage of the cells, the NOTCH signaling intensity, and the specific expression of target genes. Extensive evidence supports the notion that DLK1 and DLK2 proteins principally act by inhibiting the activity of the four NOTCH receptors. Our observations revealed an increase in the expression of all four *Notch* genes in correlation with cell confluence in culture of non-transfected cells. However, we noted a general increase in the expression of *Notch2*, *Notch3*, and *Notch4*, whereas *Notch1* expression significantly decreased in induced cells. These findings lead us to suggest that *Notch2*, *Notch3*, and *Notch4* might play essential roles in facilitating osteogenesis, while *Notch1* appears to function as an inhibitor of this differentiation process that may imply that the reduction in *Notch1* expression seems to be a crucial factor in enabling the osteogenic differentiation of these cells.

Our findings also revealed a decrease in the levels of *Notch1* during osteogenic differentiation, which suggests that the activation of the NOTCH1 receptor may inhibit osteogenesis. These observations are consistent with earlier studies that have reported that NOTCH1 activation or NICD1 overexpression inhibit osteogenic differentiation and bone extracellular matrix mineralization [39]. Previous research has also shown that DAPT’s effect on osteogenesis can vary, either promoting or inhibiting the process [40]. By using alkaline phosphatase staining method, we observed that osteoblast differentiation was inhibited in C3H10T1/2 cells in the presence of DAPT, which inhibits global NOTCH signaling. Additionally, we observed a significant decrease in the expression of all osteogenic markers in the presence of this NOTCH signaling inhibitor.

On the other hand, as cell confluence increased in non-differentiated cells, *Hes1* expression decreased, despite it being a target gene of NOTCH receptors whose expression increases with confluence. This trend reverses when cells undergo osteogenic differentiation; *Hes1* expression markedly increases, which could be due to the increase of *Notch2*, *3* and *4* expression and activation. Previous research suggests that *Hes1* expression activates RUNX2 factor, which plays a critical role in osteogenic differentiation [41]. We observed in this work that the increased *Dlk1* expression in undifferentiated cells due to cell confluence coincided with this decrease in *Hes1* expression, which suggests that DLK1 could be suppressing NOTCH signaling and *Hes1* expression. Conversely, during osteogenic induction, the decrease in *Dlk1* expression may increase *Hes1* expression and facilitate cell differentiation. This finding aligns with previous studies that reported a reciprocal inhibition between *Dlk1* and *Hes1* genes [7, 42]. The role of the *Hey1* gene in osteogenesis has been subject to contradictory findings in previous studies. *Hey1* is known to inhibit mineralization and the transcriptional activity of RUNX2 during osteogenesis [23] and its overexpression can modulate osteogenesis induced by BMP9 [43]. In our study, we observed that in both undifferentiated and differentiated C3H10T1/2 cells, *Hey1* expression levels increased compared to undifferentiated cells, which suggests that *Hey1* may be enhanced by the activity of NOTCH 2, 3, and 4 receptors in the presence of osteogenic inducers, implying its necessity in the osteogenic process.

Our findings also indicate that overexpression of DLK1 consistently reduces the expression of all osteogenic markers, as evidenced by RT-qPCR and alkaline phosphatase staining. Conversely, reduced DLK1 expression significantly enhances the expression levels of these markers. Regarding DLK2’s role, its overexpression markedly increased the expression of all osteogenic markers, but its decrease only slightly elevated the expression levels of some markers, and the alkaline phosphatase staining was comparable to control cells. These observations suggest that DLK1 acts as a potent inhibitor of osteogenic differentiation in C3H10T1/2 cells. These findings align with previous studies indicating DLK1’s inhibitory role in bone formation by suppressing RUNX2 expression [44], and influencing BMP2/Smads signaling pathways, as well as promoting osteoclastogenesis through NFκB pathway activation and proinflammatory cytokine synthesis [30]. Additionally, the protective effect of antibodies against the soluble variant of DLK1 against DLK1-induced bone loss has been documented [32]. Few studies exploring the role of DLK2 in bone cells differentiation have been published [33, 34]. Our results demonstrate, for the first time, that DLK2 appears to enhance the osteogenic process in C3H10T1/2 cells.

Additionally, our findings reveal that alterations in the expression levels of DLK1 or DLK2 proteins impact not only their own basal expression but also influence the basal expression of *Notch* and *Hes1/Hey1* genes in undifferentiated C3H10T1/2 cells. Moreover, subjecting DLK stable transfectant pools to osteogenic treatment further modulates the expression of these genes, although in varying manners. Despite being a NOTCH signaling inhibitor, overexpressing DLK1 unexpectedly increased *Hey1* expression. Additionally, a decrease in the expression of both *Dlk* genes surprisingly resulted in reduced *Hey1* expression (Fig. 3F). These observations suggest a complex interplay between the expression of NOTCH receptors and specific canonical ligands. This interplay is pivotal in regulating how precursor cells respond to external signals, thereby determining whether to initiate or not a specific differentiation processes, such as osteogenesis. These observations could also be explained by the regulation of *Hey1* expression by NOTCH independent signaling pathways.

Considering DLK1’s role as a NOTCH signaling inhibitor, it could potentially hinder osteogenesis by specifically inhibiting NOTCH2, 3, and 4 receptors, which activate osteogenic differentiation. The osteogenesis-enhancing effect of DLK2, despite it also being a NOTCH receptor inhibitor like DLK1, presents several explanatory hypotheses. One possibility is that DLK2 might counteract DLK1’s effects, as previously observed in an adipogenic context [11, 12], thereby promoting osteogenesis. Another theory is that DLK2 could selectively inhibit NOTCH1, which is thought to be inhibitory for osteogenesis, without affecting the pro-osteogenic NOTCH receptors. Alternatively, DLK2 might promote osteogenesis via a NOTCH-independent pathway, such as through the activation of the cMET receptor, as suggested in studies related to breast cancer metastasis [45]. Furthermore, recent research has shown that the deletion of DLK2 in osteoclasts significantly hampers osteoclast formation and contributes to a higher bone mass phenotype [33].

Finally, using alkaline phosphatase staining in conjunction with inhibitors of various signaling pathways, we deduced that the ERK1/2 MAPK and p38 MAPK pathways participate in the osteogenic differentiation of C3H10T1/2 cells, whereas the AKT-PI3K signaling pathway seems less implicated. Some works demonstrate that varying levels of DLK1 expression can modulate both the activity and activation kinetics of ERK1/2 MAPK [46, 47]. In the context of DLK2, its expression levels have been reported to affect ERK1/2 MAPK [48]. In this study, we observed that overexpression of DLK2 or downregulation of DLK1 may enhance ERK1/2 MAPK kinase activity, potentially promoting osteogenic differentiation. Conversely, downregulation of DLK2, but not DLK1 overexpression, could significantly inhibit ERK1/2 MAPK activity and thereby osteogenesis. This aligns with other research indicating that increased ERK1/2 MAPK phosphorylation is crucial during the osteogenic phase of matrix mineralization, as phosphorylated ERK1/2 MAPK can interact with RUNX2 and bind to promoters of late differentiation markers [49].

Based on these observations, we suggest that DLK2 overexpression, by inhibiting NOTCH1 signaling, or DLK1 downregulation, avoiding NOTCH2, 3, and 4 signaling inhibition, along with modulation of ERK1/2 MAPK activity, might create a conducive environment for osteogenic differentiation. Conversely, DLK1 may modulate NOTCH2, 3, and 4 receptors signaling and potentially inhibit ERK1/2 MAPK activity, thereby hindering osteogenesis. The involvement of p38 MAPK kinase in the osteogenic differentiation of C3H10T1/2 cells is evident from other studies [50]. In this work, we were only able to find that decreased DLK1 expression correlates with increased p38 MAPK phosphorylation during differentiation, implying that DLK1 might inhibit osteogenesis also by suppressing p38 MAPK activity. The role of DLK2 in modulating p38 MAPK seems to be dispensable.

**Fig. 8:**
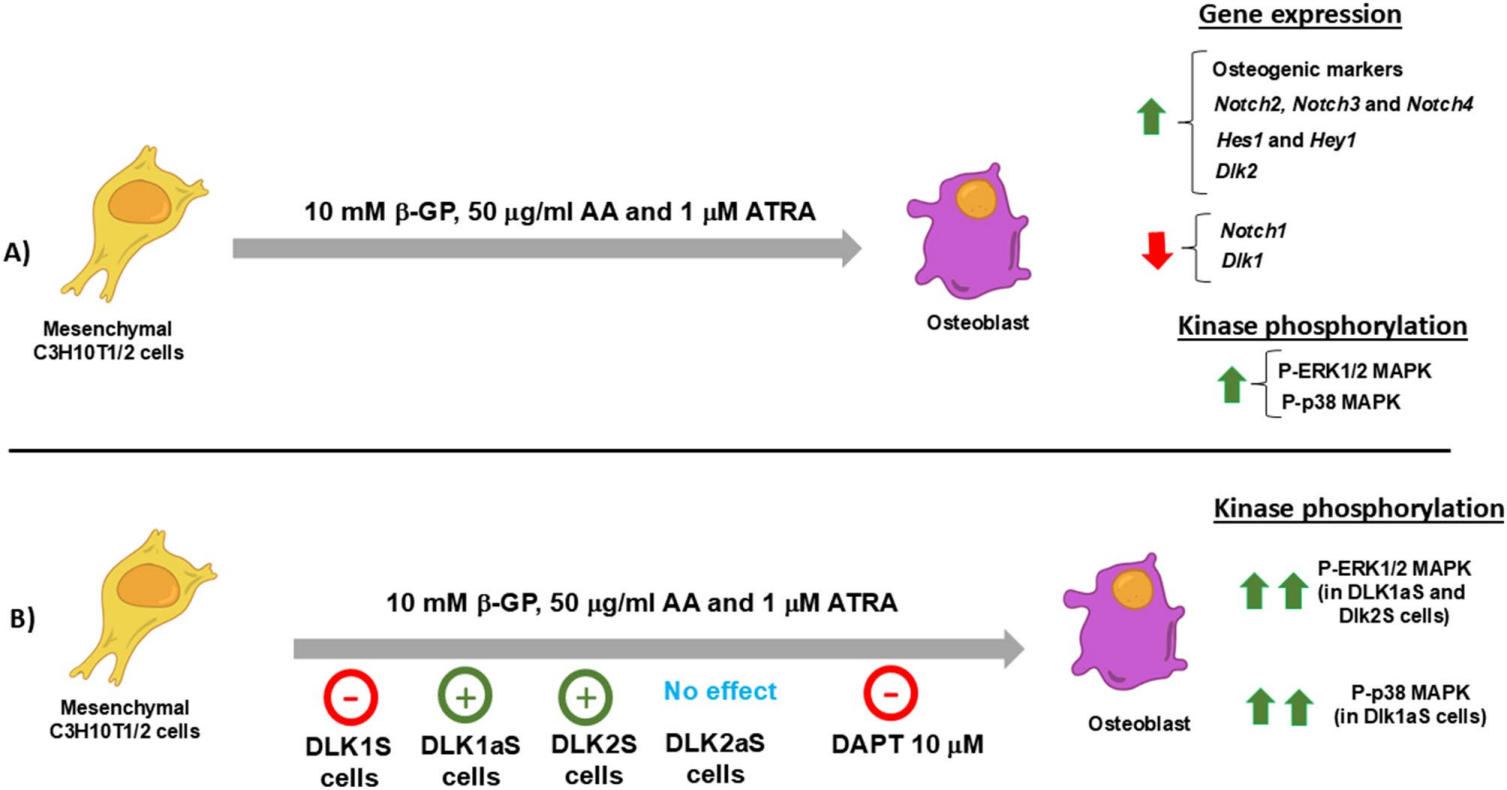
**A** Effect of osteogenic treatment (10 mM β-glycerol-phosphate (β-GP), 50 μg/ml ascorbic acid (AA), and 1 μM all-trans retinoic acid (ATRA)) on the expression levels of osteogenic markers and *Notch* family genes, as well as the phosphorylation levels of ERK1/2 and p38 MAPK, in non-transfected mesenchymal C3H10T1/2 cells. **B** Impact of treatment with DAPT, a NOTCH signaling inhibitor (GSI), on the osteogenesis of non-transfected mesenchymal C3H10T1/2 cells, and the effect of DLK1 and DLK2, two inhibitory ligands of NOTCH receptors, on the osteogenesis of mesenchymal C3H10T1/2 cells transfected with plasmids that overexpress DLK1 or DLK2 proteins (DLK1S or DLK2S: DLK1 or DLK2 in sense orientation) or plasmids that downregulate DLK1 or DLK2 proteins (DLK1aS or DLK2aS: DLK1 or DLK2 in antisense orientation). Red (-) symbol indicates inhibition of osteogenesis. Green (+) symbol indicates activation of osteogenesis. Green up arrow means increased gene expression. Red down arrow means decreased gene expression

In Fig. 8, we have summarized the effects of the osteogenic treatment and the impact of DLK1 and DLK2 protein levels on the osteogenesis of mesenchymal C3H10T1/2 cells. This work contributes novel insights into the roles of DLK proteins and the four NOTCH receptors in osteogenic differentiation. The specific modulation of a NOTCH receptor signaling by specific activating canonical ligands and inhibitory DLK proteins, coupled with the regulation of specific kinase activities involved in osteogenesis, presents potential therapeutic implications. Targeting these pathways with DLK proteins or DLK-derived peptides with or without the use of nanoparticles could offer promising strategies for treating physiological and pathological conditions like osteoporosis or bone formation.

## Materials and methods

### Plasmids, cell culture, and transfections

Plasmids pCDLK1S (DLK1S), pCDLK1aS (DLK1aS), pCDLK2S (DLK2S) and pCDLK2aS (DLK2aS) have the complete cDNA sequence of either *Dlk1* or *Dlk2* in sense or antisense orientation, respectively [9]. The pGLucWT plasmid [7, 51] was utilized to determine CSL/RBPJκ-dependent promoter activity, employing luciferase as a reporter gene. pRLTK plasmid expresses Renilla protein (Promega). pEGFP-N1 expresses GFP protein (Clontech). Plasmid pNICD1 encodes for the intracellular domain of NOTCH1 in pLNCX2-Myc vector [9]. Transformation of *Escherichia coli* TOP10 competent cells and plasmid DNA isolation and purification were performed as previously described [9]. Culture of mesenchymal C3H10T1/2 cells (C3H; ATCC CCL-226, clone 8) and their stable transfections were performed as previously described [9] by using Superfect Reagent (Qiagen) and the appropriated concentration of G418 antibiotic.

### Gene expression analysis by RT-qPCR

Confluent cell monolayers of control and transfected cells were processed to obtain total RNA by using the RNeasy Kit (Qiagen). RNA concentration was obtained by using the NanoDrop One Spectrophotometer (Thermo Scientific). cDNAs were obtained by using a cDNA synthesis kit (Fermentas) and gene expression assays were performed by RT-qPCR with the StepOne Plus RT-qPCR system (Applied Biosystems). The primers to analyze the expression levels of osteogenesis markers and other genes are described in Supplementary Table 8. *Rplp0* expression [5] was used as a control to compare the C_T_ from the different samples in RT-qPCR experiments. RT-qPCR expression analysis was repeated at least three times.

### Luciferase assays

To measure NOTCH trans-activation levels, C3H10T1/2 cell pools overexpressing each DLK protein, or C3H10T1/2 cells treated with 10 µM DAPT dissolved in DMSO, were co-transfected with pGLucWT and pRLTK plasmids. We used Renilla levels from pRLTK transfection to normalize the luciferase data. Forty-eight hours after transfection, cells were lysed and processed using the Dual-Luciferase Reporter Assay System (Promega). Luciferase signals were measured using the Orion II microplate luminometer (Berthold). Positive control cell pools were transiently transfected with pNICD1 (active NICD1). To evaluate transfection efficiency, cell pools were transiently transfected with pEGFP-N1, which expresses the GFP protein. These assays included three biological and two technical replicates.

### Protein sample preparation, electrophoresis, and Western blotting

Protein extracts from cultured cells were quantified and electrophoresed as previously described [9]. The soluble extracts were collected, and the protein concentrations were quantified. Western blotting was performed by using the appropriated dilution of primary and secondary antibodies (Supplementary Table 9). When we wanted to compare the levels of active NICD1 and NICD2 proteins among samples, we treated cells with 10 µM DAPT, an inhibitor of the γ-secretase complex (GSI).

Western blotting images were obtained by developing exposed films (CP-BU New, Agfa) with the Pierce ECL Plus Western blotting substrate kit (Thermo Scientific) in a Curix 60 developing apparatus (AGFA). Films were scanned with HP Officejet Pro 8600 scanner and protein signals were quantified by using QuantityOne 4.6.5. (Basic) software. Detection of α-Tubulin with a specific antibody (Millipore) was used as a protein loading control.

### Osteogenic differentiation assays

6000 cells/cm^2^ of the stable transfectant pools of C3H10T1/2 cells were seeded. When these cell cultures reached 70–80% confluence, osteogenic inducers were added to the culture medium. The osteogenic inducers were 10 mM β-glycerophosphate, 50 µg/ml ascorbic acid and 1 µM ATRA (all-trans retinoic acid). On days 1, 7, 14 and 21 of the osteogenic treatment, the culture medium was removed, cells were washed with DPBS and RNA and/or protein extracts were obtained. When we wanted to observe the effect of NOTCH signaling inhibition on osteogenesis, we treated cells with 10 µM DAPT. Osteogenesis assays were performed at least three times in triplicate.

### Alkaline phosphatase staining

On days 1, 7, 14 and 21 of the osteogenic treatment, osteoblasts were stained using the alkaline phosphatase method. Cells were fixed with a 1.5:1 acetone: citrate mixture, washed twice with distilled water, and incubated with a mixture of 0.2 mg/ml naphthol (Sigma) and 0.83 mg/ml Fast Red (Sigma) at a 1:1 ratio for one hour. Finally, cells were washed several times with distilled water until excess dye was removed and stored at 4 °C until imaging.

### Measurement of ALP Activity

ALP activity was determined using a colorimetric assay with p-nitrophenol phosphate (PNP) as a substrate (Sigma). Cells were washed twice with PBS and incubated with 100 µl of alkaline buffer solution and 200 µl of ALP substrate solution (5 mM PNP in alkaline buffer solution). Incubation was conducted at 37 °C for 30 min, and absorbance was measured at 405 nm. Data were normalized to cell number in each well. ALP activity is expressed as U/L.

### Statistical analysis

Data are presented as the mean ± SD of at least three different independent assays performed in triplicate. Data were analyzed with Student’s *t*-test to determine statistical significance. A *P* value of ≤ 0.05 was considered statistically significant (*); a *P* value ≤ 0.01 was considered highly statistically significant (**); and a *P* value of ≤ 0.001 was considered extremely statistically significant (***). Statistically non-significant results are shown by ns.

## Electronic Supplementary Material

Below is the link to the electronic supplementary material.

Supplementary Material 1 Figure S1. Expression analysis of *Notch* genes, their target genes, *Hes1* and *Hey1*, and *Dlk* genes in undifferentiated and differentiated C3H10T1/2 cells. This figure presents a RT-qPCR analysis of the relative mRNA expression levels of *Notch1* (A), *Notch2* (B), *Notch3* (C), *Notch4* (D), *Hes1* (E), *Hey1* (F), *Dlk1* (G), and *Dlk2* (H) in C3H10T1/2 cells. The analysis compares undifferentiated cells with those undergoing osteogenic differentiation at 1-, 7-, 14-, and 21-days post-induction. The data were normalized against the mRNA levels of the constitutive ribosomal gene *Rplp0*. The expression level for each gene is relative to its value on day 1 in undifferentiated cells, set arbitrarily at 1 [horizontal line]. The absence of the horizontal line in some graphs is due to its overlap with the horizontal axis resulting from the vertical axis scale. Results are shown as mean ± SD, derived from at least three independent assays, each conducted in triplicate. Statistical significance was determined using Student’s t-test (***p ≤ 0.001, **p ≤ 0.01, and *p ≤ 0.05), with non-significant results marked as ns.

Supplementary Material 2 Figure S2. Impact of DAPT,  a γ-secretase complex inhibitor, on NOTCH1 and NOTCH2 activation and the expression of *Hes1* and *Hey1* genes during osteogenic differentiation in C3H10T1/2 cells. Representative Western blot assays and densitometric analysis showing the expression levels of active NICD1 (A) and NICD2 (B) in C3H10T1/2 cells differentiating into osteoblasts over 1, 7, 14, and 21 days of induction, in the presence or absence of the 10 µM DAPT inhibitor. NICD1 and NICD2 levels were normalized against total NOTCH1 and NOTCH2 levels, respectively, using day 1 non-differentiated cells treated with DMSO as the baseline (horizontal line). α-Tubulin was used as a loading control. C) Global NOTCH signaling activity assessed by luciferase assay in C3H10T1/2 cells treated with 10 µM DAPT dissolved in DMSO. D) C3H10T1/2 cells transfected with pNICD1, which expresses and active form of NOTCH1, are used as a positive control of luciferase assays. Relative mRNA expression levels of *Hes1* (E) and *Hey1* (F) genes in C3H10T1/2 cells undergoing osteogenic differentiation in the presence or absence of DAPT, measured at 1, 7, 14, and 21 days of culture. RT-qPCR data were normalized against the *Rplp0* ribosomal gene, with day 1 non-differentiated cells treated with DMSO serving as the reference point (horizontal line). Expression levels are compared with values from equivalent DMSO-treated differentiated cells. Data represents mean ± SD from at least three independent assays, each in triplicate. Statistical significance was assessed using Student’s t-test (***p ≤ 0.001, **p ≤ 0.01, and *p ≤ 0.05), with ‘ns’ indicating non-significant differences.

Supplementary Material 3 Figure S3. Expression analysis of *Notch* genes and their target genes, *Hes1* and *Hey1*, in undifferentiated *Dlk* stable transfectant pools of C3H10T1/2 cells. This figure presents a RT-qPCR analysis of the relative mRNA expression levels of *Notch1* (A), *Notch2* (B), *Notch3* (C), *Notch4* (D), *Dlk2* (E), and *Dlk1* (F) in undifferentiated *Dlk1* sense (DLK1S), *Dlk1* antisense (DLK1aS), *Dlk2* sense (DLK2S) and *Dlk2* antisense (DLK2aS) stable transfectant pools of C3H10T1/2 cells. The RT-qPCR data were normalized against the ribosomal gene *Rplp0*, with expression levels calculated relative to day 1 in cells stably transfected with the empty vector (set arbitrarily at 1) [horizontal line]. The absence of the horizontal line in some graphs is due to its overlap with the horizontal axis due to scale adjustments. Data are shown as mean ± SD from at least three independent assays, each performed in triplicate. Statistical significance was evaluated using Student’s t-test (***p ≤ 0.001, **p ≤ 0.01, and *p ≤ 0.05), and non-significant results are indicated as ns.

Supplementary Material 4 Figure S4. Expression of *Notch* genes and their target genes, *Hes1* and *Hey1*, in *Dlk1* stable transfectant pools of C3H10T1/2 cells during osteoblast differentiation. This figure displays a RT-qPCR analysis of the relative mRNA expression levels of *Notch1* (A), *Notch2* (B), *Notch3* (C), *Notch4* (D), *Hes1* (E), and *Hey1* (F) in *Dlk1* sense (DLK1S) and antisense (DLK1aS) stable transfectant pools of C3H10T1/2 cells differentiated into osteoblasts. The analyses were conducted at 1-, 7-, 14-, and 21-days [D] post-induction of osteogenic differentiation. The RT-qPCR data were normalized against the mRNA expression levels of the ribosomal gene *Rplp0*, and the expression levels of each marker were calculated relative to day 1 in cells stably transfected with the empty vector (set arbitrarily at 1) [horizontal line]. The absence of the horizontal line in some graphs is a result of its coincidence with the horizontal axis due to the scaling of the vertical axis. Data are presented as mean ± SD from at least three independent assays, each performed in triplicate. Statistical significance was determined using Student’s t-test (***p ≤ 0.001, **p ≤ 0.01, and * *p* ≤ 0.05), and non-significant results are denoted as ns.

Supplementary Material 5 Figure S5. Expression of *Notch* genes and their target genes, *Hes1* and *Hey1*, in *Dlk2* stable transfectant pools of C3H10T1/2 cells during osteoblast differentiation. This figure depicts a RT-qPCR analysis of the relative mRNA expression levels of *Notch1* (A), *Notch2* (B), *Notch3* (C), *Notch4* (D), *Hes1* (E), and *Hey* (F) in *Dlk2* sense (DLK2S) and antisense (DLK2aS) stable transfectant pools of C3H10T1/2 cells differentiated into osteoblasts. The analyses were conducted at 1-, 7-, 14-, and 21-days [D] post-induction of osteogenic differentiation. The RT-qPCR data were normalized against the mRNA expression levels of the constitutive ribosomal gene *Rplp0*. The expression level of each marker was compared with the value obtained in non-differentiated cells stably transfected with the empty vector on day 1, represented by a horizontal line in the graphs. Data are presented as the mean ± standard deviation (SD) of at least three assays, each performed in triplicate. Statistical significance of the results for each stable transfectant was determined using Student’s t-test (***p ≤ 0.001, **p ≤ 0.01, and * p ≤ 0.05). Non-significant results are marked as ns.

Supplementary Material 6 Figure S6. Alkaline phosphatase staining of C3H10T1/2 cell cultures undergoing osteoblastic differentiation in the presence of kinase inhibitors. This figure showcases representative images of C3H10T1/2 cell cultures, as they differentiate into osteoblasts in the presence of various kinase inhibitors. The inhibitors used include U0126 (an ERK1/2 MAPK inhibitor), rapamycin (a mTOR inhibitor), LY294002 (a PI3K/AKT inhibitor), and SB203580 (a p38 MAPK inhibitor), along with DMSO-treated cells serving as control. The cells were stained using the alkaline phosphatase method at intervals of 1-, 7-, 14-, and 21-days post-induction of osteogenic differentiation. These images provide a comparative view of the effects of different kinase inhibitors on osteoblastic differentiation in C3H10T1/2 cells, demonstrating the diverse roles these kinases play in the osteogenic process.

Supplementary Material 7 Figure S7. Analysis of ERK1/2 and p38 MAPK kinase phosphorylation levels in differentiated C3H10T1/2 cells. This figure presents representative Western blots (left) and densitometric analyses (right) highlighting the phosphorylation levels of ERK1/2 MAPK (P-ERK1/2 MAPK) and p38 MAPK (P-p38) in C3H10T1/2 cells differentiated into osteoblasts. The analysis encompasses four time points post-induction of osteogenic differentiation: 1, 7, 14, and 21 days [D]. The phosphorylation levels are relative to those observed on day 1 in undifferentiated cells, set as a baseline (denoted by a horizontal line). Total ERK1/2 MAPK and total p38 MAPK expression levels were employed as controls for loading and sample quality. The densitometric data depicted in the graphs represent the mean ± standard deviation (SD) from a minimum of three independent experiments, each performed in triplicate. Statistical significance of the observed changes in phosphorylation levels at each time point was determined using Student’s t-test, with significance indicated as ***p ≤ 0.001, **p ≤ 0.01, and * p ≤ 0.05. Non-significant results are marked as ns. These analyses provide crucial insights into the temporal dynamics of ERK1/2 MAPK and p38 MAPK activation during the osteogenic differentiation process in C3H10T1/2 cells, underscoring the roles these kinases play in cellular maturation and bone formation.

Supplementary Material 8

Supplementary Material 9

## Data Availability

All data generated or analysed during this study are included in this published article [and its supplementary information files].
