# Supplementary figures and images for: DLK1 and DLK2, two non-canonical ligands of NOTCH receptors, differentially modulate the osteogenic differentiation of mesenchymal C3H10T1/2 cells
Source: Biol Res. 2024 Oct 30;57:77. doi: 10.1186/s40659-024-00561-7 (PMC11523663; doi:10.1186/s40659-024-00561-7)

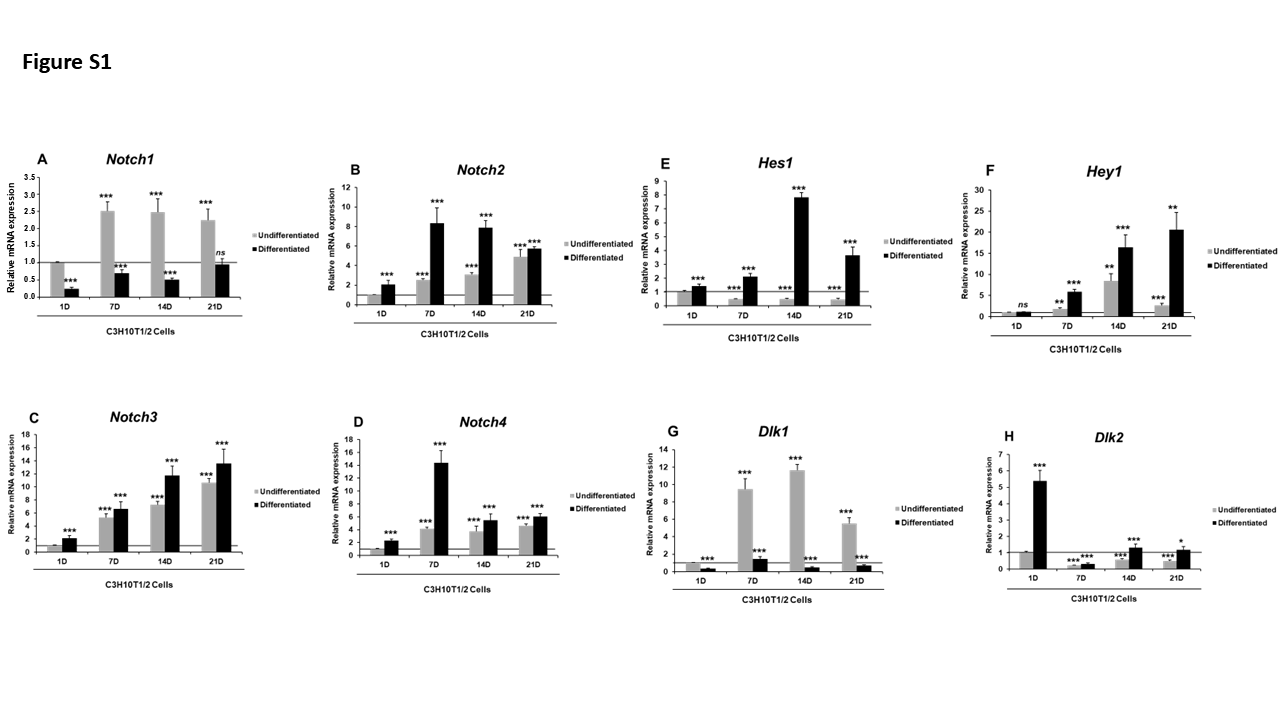

Supplement: Supplementary file 1 — Supplementary Material 1 Figure S1. Expression analysis of Notch genes, their target genes, Hes1 and Hey1, and Dlk genes in undifferentiated and differentiated C3H10T1/2 cells. This figure presents a RT-qPCR analysis of the relative mRNA expression levels of Notch1 (A), Notch2 (B), Notch3 (C), Notch4 (D), Hes1 (E), Hey1 (F), Dlk1 (G), and Dlk2 (H) in C3H10T1/2 cells. The analysis compares undifferentiated cells with those undergoing osteogenic differentiation at 1-, 7-, 14-, and 21-days post-induction. The data were normalized against the mRNA levels of the constitutive ribosomal gene Rplp0. The expression level for each gene is relative to its value on day 1 in undifferentiated cells, set arbitrarily at 1 [horizontal line]. The absence of the horizontal line in some graphs is due to its overlap with the horizontal axis resulting from the vertical axis scale. Results are shown as mean ± SD, derived from at least three independent assays, each conducted in triplicate. Statistical significance was determined using Student’s t-test (***p ≤ 0.001, **p ≤ 0.01, and *p ≤ 0.05), with non-significant results marked as ns. [file 40659_2024_561_MOESM1_ESM.tif]

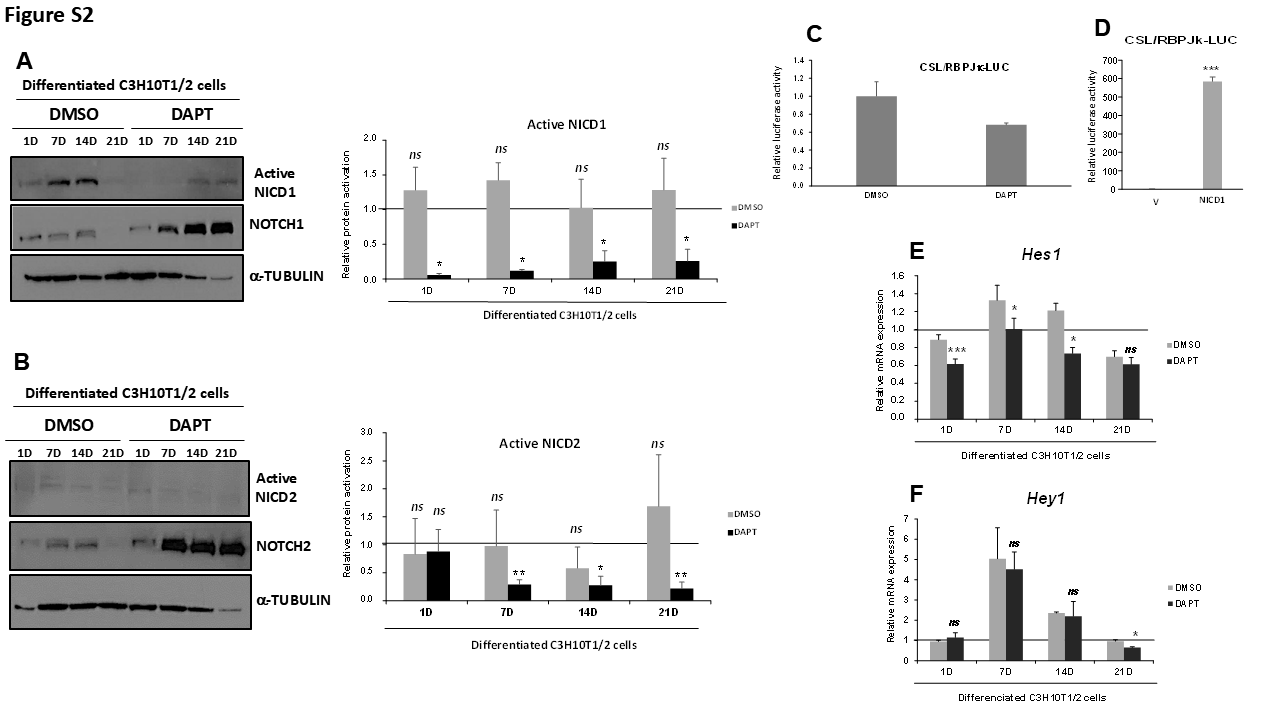

Supplement: Supplementary file 2 — Supplementary Material 2 Figure S2. Impact of DAPT, a γ-secretase complex inhibitor, on NOTCH1 and NOTCH2 activation and the expression of Hes1 and Hey1 genes during osteogenic differentiation in C3H10T1/2 cells. Representative Western blot assays and densitometric analysis showing the expression levels of active NICD1 (A) and NICD2 (B) in C3H10T1/2 cells differentiating into osteoblasts over 1, 7, 14, and 21 days of induction, in the presence or absence of the 10 µM DAPT inhibitor. NICD1 and NICD2 levels were normalized against total NOTCH1 and NOTCH2 levels, respectively, using day 1 non-differentiated cells treated with DMSO as the baseline (horizontal line). α-Tubulin was used as a loading control. C) Global NOTCH signaling activity assessed by luciferase assay in C3H10T1/2 cells treated with 10 µM DAPT dissolved in DMSO. D) C3H10T1/2 cells transfected with pNICD1, which expresses and active form of NOTCH1, are used as a positive control of luciferase assays. Relative mRNA expression levels of Hes1 (E) and Hey1 (F) genes in C3H10T1/2 cells undergoing osteogenic differentiation in the presence or absence of DAPT, measured at 1, 7, 14, and 21 days of culture. RT-qPCR data were normalized against the Rplp0 ribosomal gene, with day 1 non-differentiated cells treated with DMSO serving as the reference point (horizontal line). Expression levels are compared with values from equivalent DMSO-treated differentiated cells. Data represents mean ± SD from at least three independent assays, each in triplicate. Statistical significance was assessed using Student’s t-test (***p ≤ 0.001, **p ≤ 0.01, and *p ≤ 0.05), with ‘ns’ indicating non-significant differences. [file 40659_2024_561_MOESM2_ESM.tif]

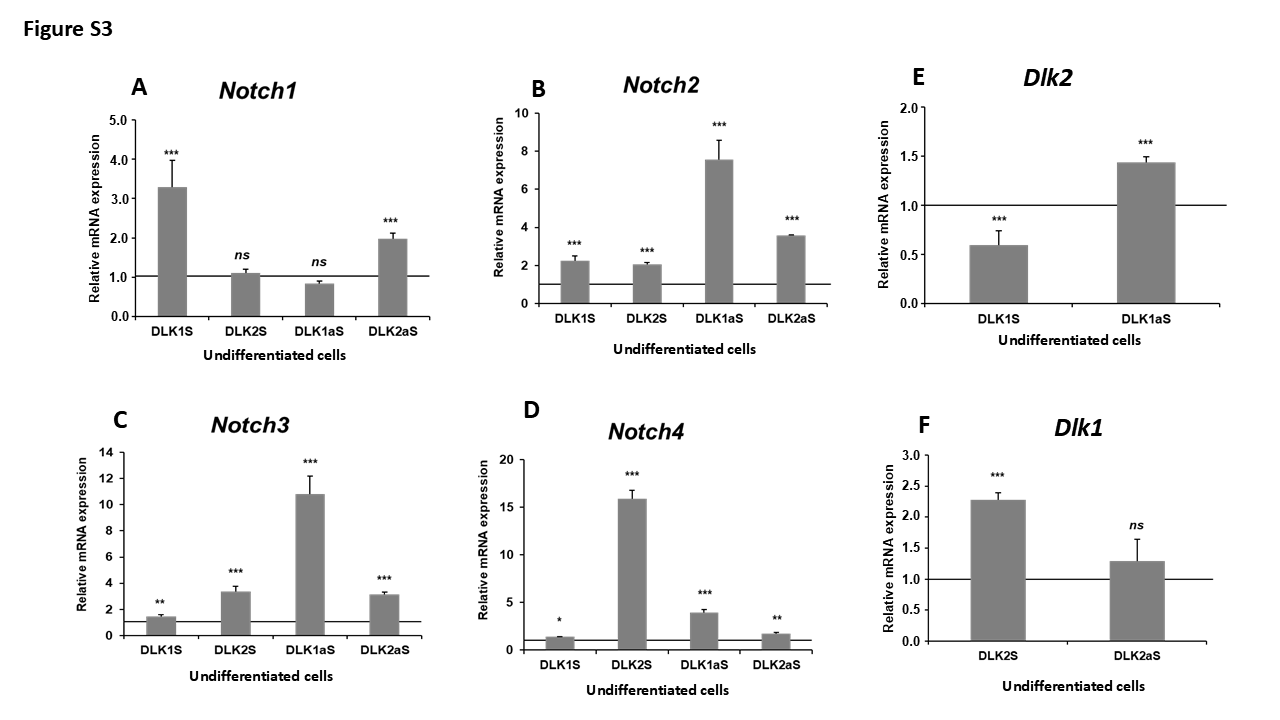

Supplement: Supplementary file 3 — Supplementary Material 3 Figure S3. Expression analysis of Notch genes and their target genes, Hes1 and Hey1, in undifferentiated Dlk stable transfectant pools of C3H10T1/2 cells. This figure presents a RT-qPCR analysis of the relative mRNA expression levels of Notch1 (A), Notch2 (B), Notch3 (C), Notch4 (D), Dlk2 (E), and Dlk1 (F) in undifferentiated Dlk1 sense (DLK1S), Dlk1 antisense (DLK1aS), Dlk2 sense (DLK2S) and Dlk2 antisense (DLK2aS) stable transfectant pools of C3H10T1/2 cells. The RT-qPCR data were normalized against the ribosomal gene Rplp0, with expression levels calculated relative to day 1 in cells stably transfected with the empty vector (set arbitrarily at 1) [horizontal line]. The absence of the horizontal line in some graphs is due to its overlap with the horizontal axis due to scale adjustments. Data are shown as mean ± SD from at least three independent assays, each performed in triplicate. Statistical significance was evaluated using Student’s t-test (***p ≤ 0.001, **p ≤ 0.01, and *p ≤ 0.05), and non-significant results are indicated as ns. [file 40659_2024_561_MOESM3_ESM.tif]

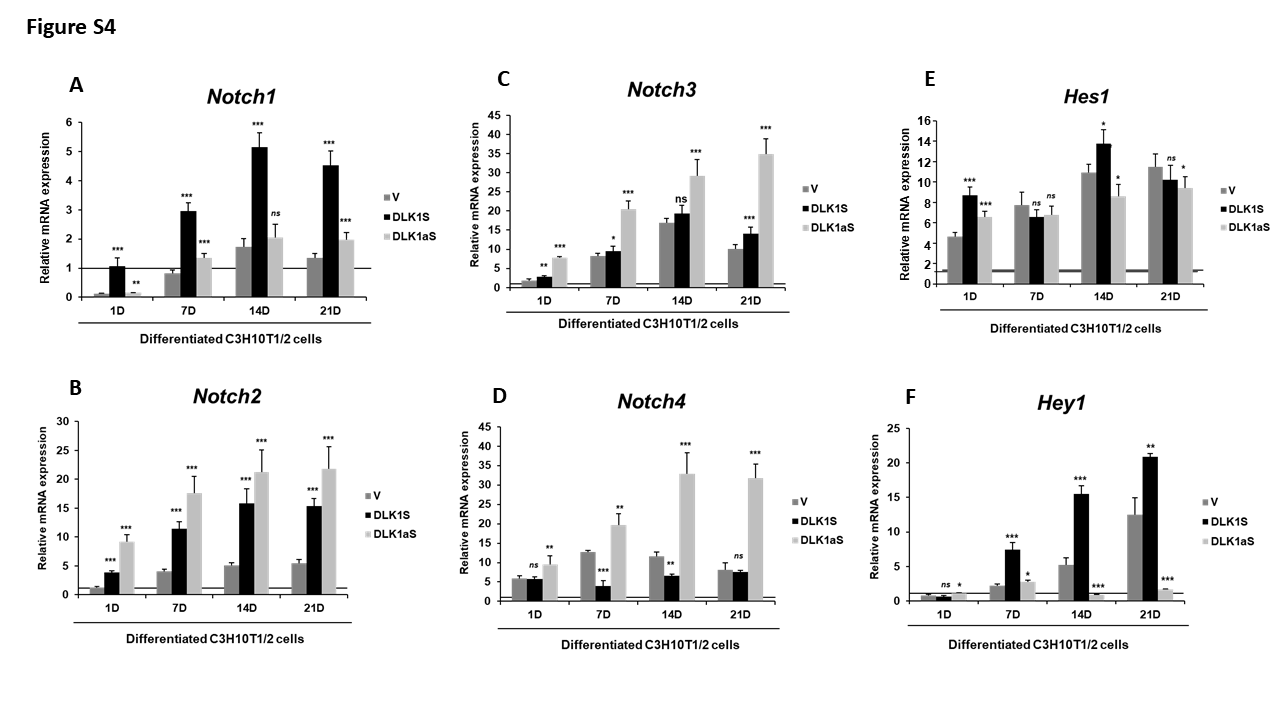

Supplement: Supplementary file 4 — Supplementary Material 4 Figure S4. Expression of Notch genes and their target genes, Hes1 and Hey1, in Dlk1 stable transfectant pools of C3H10T1/2 cells during osteoblast differentiation. This figure displays a RT-qPCR analysis of the relative mRNA expression levels of Notch1 (A), Notch2 (B), Notch3 (C), Notch4 (D), Hes1 (E), and Hey1 (F) in Dlk1 sense (DLK1S) and antisense (DLK1aS) stable transfectant pools of C3H10T1/2 cells differentiated into osteoblasts. The analyses were conducted at 1-, 7-, 14-, and 21-days [D] post-induction of osteogenic differentiation. The RT-qPCR data were normalized against the mRNA expression levels of the ribosomal gene Rplp0, and the expression levels of each marker were calculated relative to day 1 in cells stably transfected with the empty vector (set arbitrarily at 1) [horizontal line]. The absence of the horizontal line in some graphs is a result of its coincidence with the horizontal axis due to the scaling of the vertical axis. Data are presented as mean ± SD from at least three independent assays, each performed in triplicate. Statistical significance was determined using Student’s t-test (***p ≤ 0.001, **p ≤ 0.01, and * p ≤ 0.05), and non-significant results are denoted as ns. [file 40659_2024_561_MOESM4_ESM.tif]

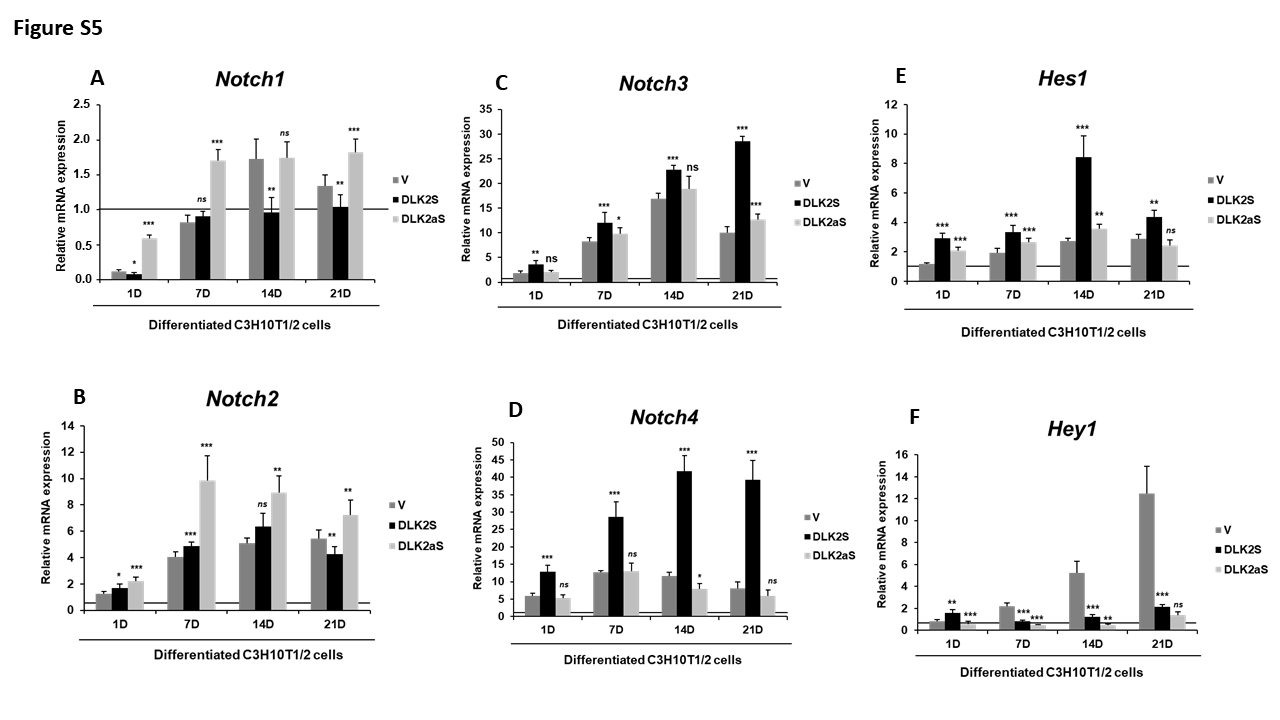

Supplement: Supplementary file 5 — Supplementary Material 5 Figure S5. Expression of Notch genes and their target genes, Hes1 and Hey1, in Dlk2 stable transfectant pools of C3H10T1/2 cells during osteoblast differentiation. This figure depicts a RT-qPCR analysis of the relative mRNA expression levels of Notch1 (A), Notch2 (B), Notch3 (C), Notch4 (D), Hes1 (E), and Hey (F) in Dlk2 sense (DLK2S) and antisense (DLK2aS) stable transfectant pools of C3H10T1/2 cells differentiated into osteoblasts. The analyses were conducted at 1-, 7-, 14-, and 21-days [D] post-induction of osteogenic differentiation. The RT-qPCR data were normalized against the mRNA expression levels of the constitutive ribosomal gene Rplp0. The expression level of each marker was compared with the value obtained in non-differentiated cells stably transfected with the empty vector on day 1, represented by a horizontal line in the graphs. Data are presented as the mean ± standard deviation (SD) of at least three assays, each performed in triplicate. Statistical significance of the results for each stable transfectant was determined using Student’s t-test (***p ≤ 0.001, **p ≤ 0.01, and * p ≤ 0.05). Non-significant results are marked as ns. [file 40659_2024_561_MOESM5_ESM.tif]

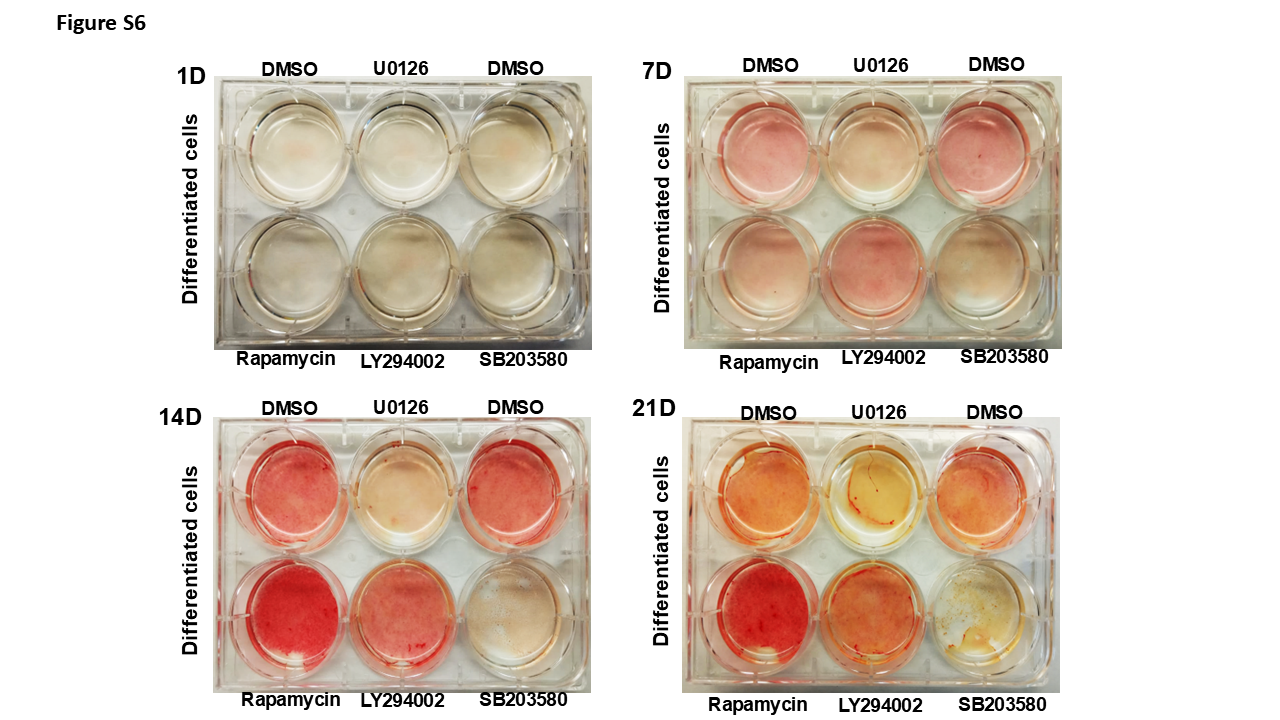

Supplement: Supplementary file 6 — Supplementary Material 6 Figure S6. Alkaline phosphatase staining of C3H10T1/2 cell cultures undergoing osteoblastic differentiation in the presence of kinase inhibitors. This figure showcases representative images of C3H10T1/2 cell cultures, as they differentiate into osteoblasts in the presence of various kinase inhibitors. The inhibitors used include U0126 (an ERK1/2 MAPK inhibitor), rapamycin (a mTOR inhibitor), LY294002 (a PI3K/AKT inhibitor), and SB203580 (a p38 MAPK inhibitor), along with DMSO-treated cells serving as control. The cells were stained using the alkaline phosphatase method at intervals of 1-, 7-, 14-, and 21-days post-induction of osteogenic differentiation. These images provide a comparative view of the effects of different kinase inhibitors on osteoblastic differentiation in C3H10T1/2 cells, demonstrating the diverse roles these kinases play in the osteogenic process. [file 40659_2024_561_MOESM6_ESM.tif]

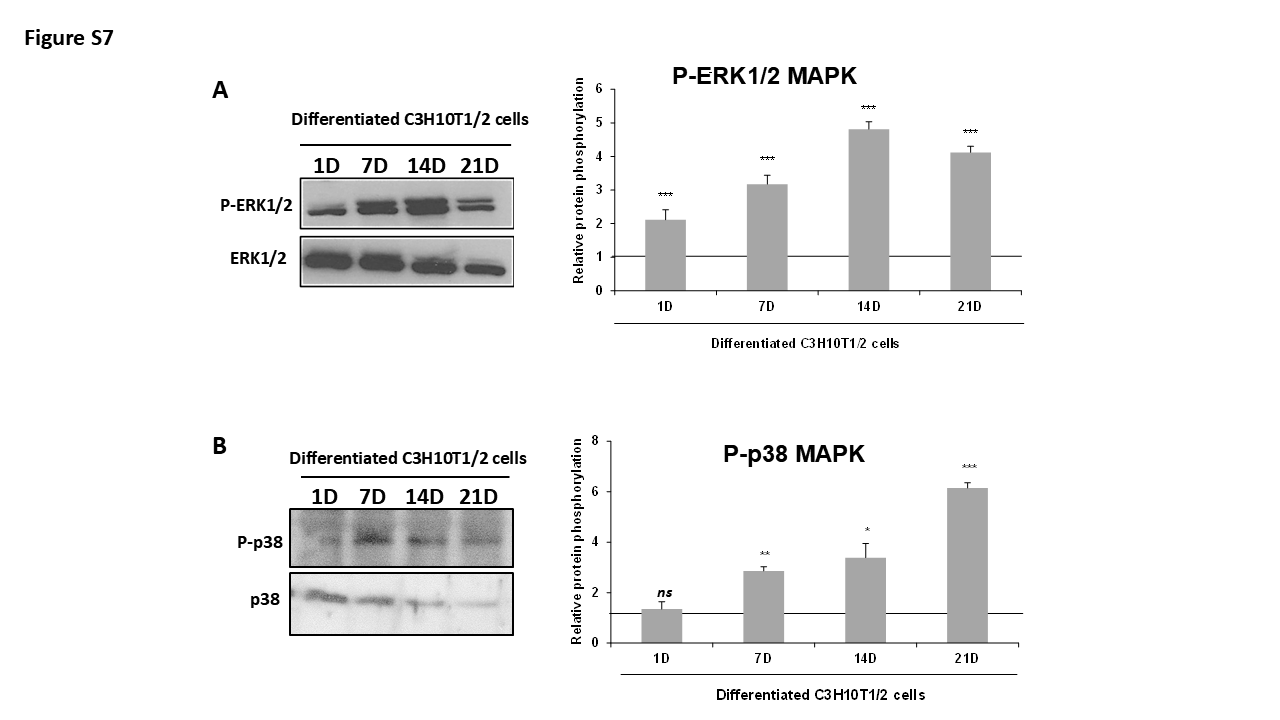

Supplement: Supplementary file 7 — Supplementary Material 7 Figure S7. Analysis of ERK1/2 and p38 MAPK kinase phosphorylation levels in differentiated C3H10T1/2 cells. This figure presents representative Western blots (left) and densitometric analyses (right) highlighting the phosphorylation levels of ERK1/2 MAPK (P-ERK1/2 MAPK) and p38 MAPK (P-p38) in C3H10T1/2 cells differentiated into osteoblasts. The analysis encompasses four time points post-induction of osteogenic differentiation: 1, 7, 14, and 21 days [D]. The phosphorylation levels are relative to those observed on day 1 in undifferentiated cells, set as a baseline (denoted by a horizontal line). Total ERK1/2 MAPK and total p38 MAPK expression levels were employed as controls for loading and sample quality. The densitometric data depicted in the graphs represent the mean ± standard deviation (SD) from a minimum of three independent experiments, each performed in triplicate. Statistical significance of the observed changes in phosphorylation levels at each time point was determined using Student’s t-test, with significance indicated as ***p ≤ 0.001, **p ≤ 0.01, and * p ≤ 0.05. Non-significant results are marked as ns. These analyses provide crucial insights into the temporal dynamics of ERK1/2 MAPK and p38 MAPK activation during the osteogenic differentiation process in C3H10T1/2 cells, underscoring the roles these kinases play in cellular maturation and bone formation. [file 40659_2024_561_MOESM7_ESM.tif]

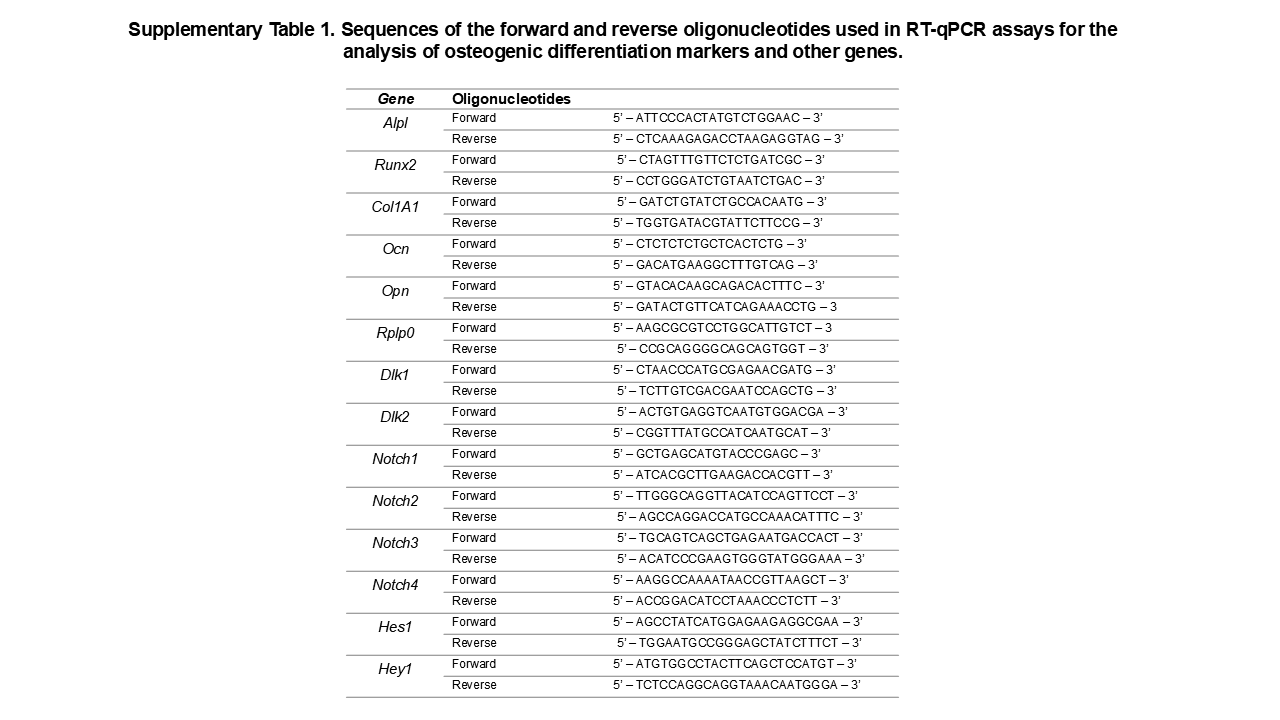

Supplement: Supplementary file 8 — Supplementary Material 8 [file 40659_2024_561_MOESM8_ESM.tif]

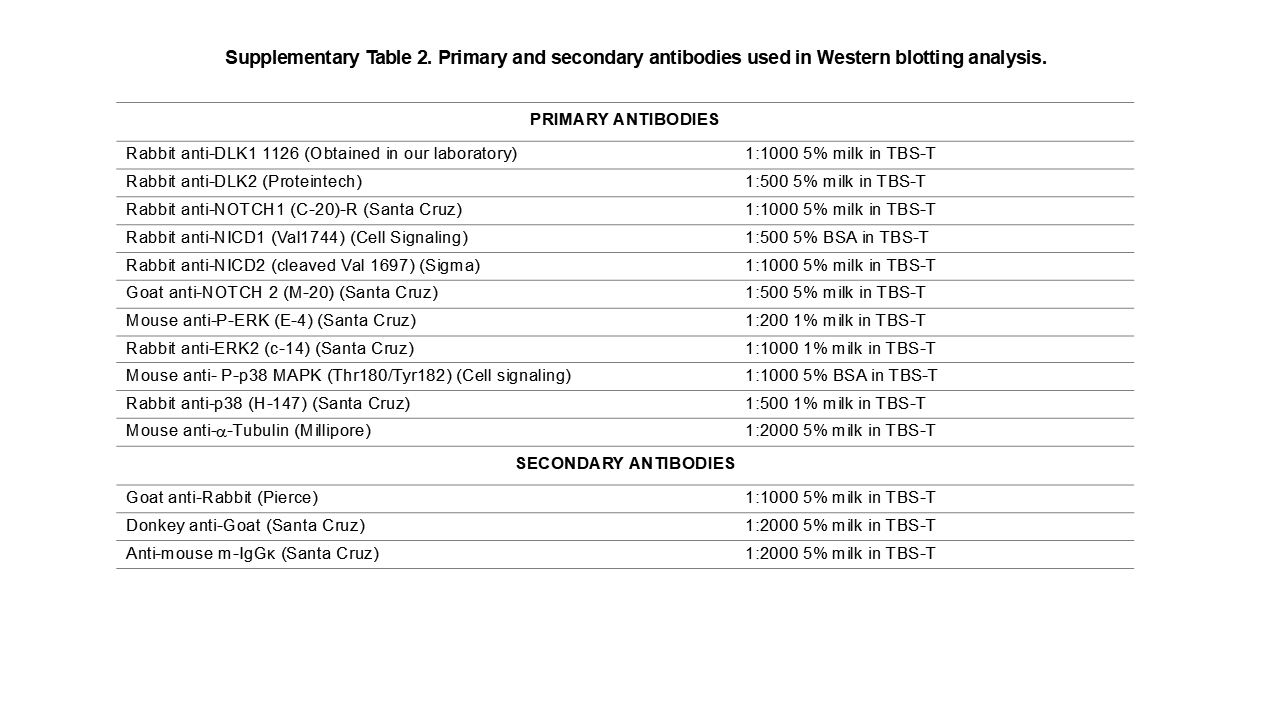

Supplement: Supplementary file 9 — Supplementary Material 9 [file 40659_2024_561_MOESM9_ESM.tif]
